# Supplementary material for: An adaptable implementation package targeting evidence-based indicators in primary care: A pragmatic cluster-randomised evaluation
Source: PLoS Med. 2020 Feb 28;17(2):e1003045. doi: 10.1371/journal.pmed.1003045 (PMC7048270; doi:10.1371/journal.pmed.1003045)
Supplement: S1 Table — All adjusted for covariates and baseline achievement of primary outcomes. Table presents mean percentage achievement, unless otherwise stated. Variables controlled for in the adjusted analyses were as follows: patient-level sex and age, and practice-level baseline list size, CCG, pre-intervention achievement against primary outcomes, total QOF score 2014–2015, and proportion of patients with 0–3 comorbidities. ACE-I, angiotensin-converting-enzyme inhibitor; ACR: albumin:creatinine ratio; ARB, angiotensin receptor blocker; BMI, body mass index; BP, blood pressure; CCG, clinical commissioning group; CI, confidence interval; CKD, chronic kidney disease; eGFR, estimated glomerular filtration rate; HbA1c, haemoglobin A1c; NSAID, non-steroidal anti-inflammatory drug; PCR, protein:creatinine ratio; QOF, Quality Outcomes Framework. (DOCX) [file pmed.1003045.s001.docx]

**Supplementary Table 1. Secondary outcomes from Trial 1: Achievement of individual indicators that contributed to composite outcomes; processes of care; continuous intermediate clinical outcomes. All adjusted for covariates and baseline achievement of primary outcomes. Table presents mean % achievement, unless otherwise stated.**

|  | Unadjusted model estimates | | | | Adjusted model estimates | | | |
| --- | --- | --- | --- | --- | --- | --- | --- | --- |
|  | Diabetes control (n=40; %) | Risky prescribing (n=40; %) | Odds ratio (97.5% CI) | p-value | Diabetes control (n=40; %) | Risky prescribing (n=40; %) | Odds ratio (97.5% CI) | p-value |
| *Type 2 diabetes indicators that contributed to the composite outcome*  Patients with type 2 diabetes (indicators refer to preceding 12 months) | | | | | | | | |
| Last recorded BP <140/80 mmHg (or <130/80 mmHg if complications*) | 45.8 | 45.5 | 1.014 (0.841, 1.222) | 0.868 | 45.6 | 45.0 | 1.024 (0.844, 1.243) | 0.780 |
| Last recorded HbA1c ≤ 59 mmol/mol | 67.5 | 67.0 | 1.025 (0.868, 1.211) | 0.736 | 69.1 | 68.0 | 1.051 (0.934, 1.182) | 0.344 |
| Last recorded cholesterol ≤ 5 mmol/l | 72.5 | 73.3 | 0.959 (0.824, 1.117) | 0.540 | 72.1 | 73.8 | 0.921 (0.788, 1.076) | 0.234 |
| *Diabetes processes of care*  Patients with type 2 diabetes (indicators refer to preceding 12 months) | | | | | | | | |
| Record of BP measurement | 95.9 | 96.1 | 0.952 (0.704, 1.285) | 0.711 | 96.5 | 96.5 | 0.987 (0.731, 1.331) | 0.920 |
| Record of HbA1c measurement | 95.1 | 95.3 | 0.957 (0.692, 1.322) | 0.759 | 95.5 | 95.6 | 0.982 (0.732, 1.316) | 0.888 |
| Record of total cholesterol measurement | 89.6 | 89.4 | 1.022 (0.782, 1.337) | 0.853 | 89.4 | 89.5 | 0.993 (0.772, 1.276) | 0.949 |
| Record of urine ACR or PCR measurement or proteinuria code | 64.8 | 59.0 | 1.278 (0.961, 1.699) | 0.054 | 64.1 | 58.9 | 1.248 (0.922, 1.690) | 0.102 |
| Record of eGFR or serum creatinine measurement | 93.2 | 92.8 | 1.060 (0.797, 1.410) | 0.646 | 93.9 | 93.4 | 1.079 (0.811, 1.435) | 0.551 |
| Record of foot review | 83.9 | 80.9 | 1.231 (0.867, 1.747) | 0.183 | 85.0 | 82.5 | 1.201 (0.853, 1.691) | 0.229 |
| Record of retinal screening | 76.2 | 73.7 | 1.140 (0.719, 1.807) | 0.524 | 77.2 | 75.9 | 1.071 (0.737, 1.557) | 0.681 |
| Record of BMI measurement | 85.6 | 84.3 | 1.107 (0.678, 1.807) | 0.641 | 86.5 | 83.4 | 1.271 (0.831, 1.942) | 0.206 |
| Record of smoking status | 86.9 | 84.5 | 1.212 (0.779, 1.888) | 0.330 | 87.4 | 83.3 | 1.400 (0.940, 2.086) | 0.059 |
| All above processes | 35.9 | 30.2 | 1.294 (0.861, 1.946) | 0.156 | 36.2 | 31.0 | 1.260 (0.865, 1.836) | 0.168 |
| All above processes, excluding retinal screening | 45.7 | 39.1 | 1.311 (0.923, 1.863) | 0.084 | 45.4 | 38.5 | 1.324 (0.942, 1.862) | 0.065 |
|  | Diabetes control (n=40; mean) | Risky prescribing (n=40; mean) | Difference (97.5% CI) | p-value | Diabetes control (n=40; mean) | Risky prescribing (n=40; mean) | Difference (97.5% CI) | p-value |
| *Continuous intermediate clinical outcomes*  Last recorded value in previous 12 months | | | | | | | | |
| Systolic blood pressure (mmHg) | 131.6 | 131.7 | -0.101 (-1.488, 1.286) | 0.870 | 131.7 | 131.7 | -0.036 (-1.490, 1.418) | 0.956 |
| Diastolic blood pressure (mmHg) | 74.9 | 75.2 | -0.288 (-1.211, 0.636) | 0.485 | 74.8 | 75.0 | -0.247 (-1.261, 0.766) | 0.584 |
| HbA1c^†^ (mmol/mol) | 56.5 | 56.5 | -0.002 (-0.025, 0.022) | 0.881 | 56.0 | 56.1 | -0.003 (-0.019, 0.012) | 0.655 |
| Total serum cholesterol^†^ (mmol/l) | 4.1 | 4.1 | 0.003 (-0.013, 0.018) | 0.684 | 4.2 | 4.1 | 0.009 (-0.007, 0.025) | 0.193 |
|  | Diabetes control (n=40; %) | Risky prescribing (n=40; %) | Odds ratio (97.5% CI) | p-value | Diabetes control (n=40; %) | Risky prescribing (n=40; %) | Odds ratio (97.5% CI) | p-value |
| *Risky prescribing indicators that contributed to the composite outcome* | | | | | | | | |
| 1. NSAID^†^ or low-dose aspirin prescribed in previous 8 weeks and not prescribed gastro-protection in previous 12 weeks | 26.6 | 24.9 | 0.916 (0.506, 1.658) | 0.739 | 21.6 | 20.8 | 0.953 (0.488, 1.862) | 0.872 |
| 2. Aged ≥75y and prescribed NSAID^†^ in previous 8 weeks and not prescribed gastro-protection in previous 12 weeks | 28.6 | 26.3 | 0.889 (0.580, 1.364) | 0.538 | 26.9 | 28.4 | 1.075 (0.683, 1.693) | 0.721 |
| 3. Aged ≥65y and prescribed aspirin in previous 12 weeks and prescribed NSAID^†^ in previous 8 weeks and not prescribed gastro-protection in previous 12 weeks | 23.8 | 21.3 | 0.870 (0.478, 1.583) | 0.601 | 18.8 | 20.8 | 1.133(0.596, 2.153) | 0.662 |
| 4. Aged ≥65y and prescribed aspirin in previous 12 weeks and prescribed clopidogrel in previous 8 weeks and not prescribed gastro-protection in previous 12 weeks | 36.1 | 27.3 | 0.666 (0.451, 0.984) | 0.019 | 35.2 | 25.3 | 0.624 (0.395, 0.987) | 0.021 |
| 5. Prescribed warfarin in previous 12 weeks and prescribed NSAID^†^ in previous 12 weeks | 1.2 | 1.1 | 0.894 (0.497, 1.607) | 0.668 | 1.0 | 0.9 | 0.885 (0.467, 1.678) | 0.669 |
| 6. Prescribed warfarin in previous 12 weeks and low-dose aspirin or clopidogrel in previous 8 weeks and not prescribed gastro-protection in previous 12 weeks | 38.2 | 37.4 | 0.964 (0.514, 1.810) | 0.896 | 36.9 | 35.7 | 0.948 (0.478, 1.880) | 0.861 |
| 7. Record of heart failure and prescribed any oral NSAID in previous 8 weeks | 2.8 | 2.0 | 0.710 (0.399, 1.260) | 0.181 | 2.1 | 1.6 | 0.752 (0.430, 1.316) | 0.254 |
| 8. Prescribed diuretic and ACE-I or ARB in previous 12 weeks, and prescribed any oral NSAID in previous 8 weeks | 5.7 | 4.5 | 0.784 (0.559, 1.099) | 0.106 | 5.0 | 4.0 | 0.788 (0.612, 1.015) | 0.035 |
| 9. CKD and prescribed any oral NSAID in previous 8 weeks | 3.5 | 3.0 | 0.856 (0.617, 1.187) | 0.286 | 3.0 | 2.7 | 0.916 (0.698, 1.204) | 0.473 |
| Gastro-intestinal composite. The proportion of patients achieving at least one of indicators 1-6 (above) | 12.2 | 9.7 | 0.778 (0.544, 1.113) | 0.116 | 10.9 | 9.3 | 0.843 (0.648, 1.097) | 0.146 |
| Renal composite. The proportion of patients achieving at least one of indicators 8 and 9 (above) | 4.8 | 3.9 | 0.817 (0.597, 1.117) | 0.148 | 4.1 | 3.4 | 0.823 (0.656, 1.033) | 0.054 |

CI = confidence interval; BP = blood pressure; HbA1c = haemoglobin A1c; ACR: albumin:creatinine ratio; PCR = protein:creatinine ratio; eGFR = estimated glomerular filtration rate; BMI = body mass index; NSAID = non-steroidal anti-inflammatory drug; ACE-I = angiotensin-converting-enzyme inhibitor; ARB = angiotensin receptor blocker; CKD = chronic kidney disease

Variables controlled for in the adjusted analyses were: patient-level sex and age, and practice-level baseline list size, CCG, pre-intervention achievement against primary outcomes, total QOF score 2014-15, and proportion of patients with 0-3 comorbidities
