# Supplementary material for: An adaptable implementation package targeting evidence-based indicators in primary care: A pragmatic cluster-randomised evaluation
Source: PLoS Med. 2020 Feb 28;17(2):e1003045. doi: 10.1371/journal.pmed.1003045 (PMC7048270; doi:10.1371/journal.pmed.1003045)
Supplement: S2 Table — All adjusted for covariates and baseline achievement of primary outcomes. Note: Formal statistical testing was inappropriate due to violation of the modelling assumptions for the following trial-related indicators: DM006, DM014, DM018; and the following non-trial related indicators: CHD005, CHD007, MH002, MH003, SMOK004, and SMOK005. Summary statistics only are presented for these indicators. †The HbA1c and total serum cholesterol continuous intermediate clinical outcomes were analysed using a log transformation in order to satisfy the modelling assumptions. The predicted means presented are on the untransformed (original) scale, but the estimated intervention effect (and 97.5% CI) are on the log scale. *If urine albumin:creatinine ratio ≥3, or retinopathy, or record of cerebrovascular accident or transient ischemic attack. Variables controlled for in the adjusted analyses were as follows: practice-level baseline list size, CCG, pre-intervention achievement against primary outcomes, and total QOF score 2014–2015. ACE-I, angiotensin-converting-enzyme inhibitor; ARB, angiotensin receptor blocker; CCG, clinical commissioning group; CHD, coronary heart disease; CI, confidence interval; CKD, chronic kidney disease (stage 3–5); COPD, chronic obstructive pulmonary disease; HbA1c, haemoglobin A1c; IFCC, International Federation of Clinical Chemistry and Laboratory Medicine; PAD, peripheral arterial disease; QOF, quality and outcomes framework; RCP, Royal College of Physicians; TIA, transient ischemic attack. (DOCX) [file pmed.1003045.s002.docx]

**Supplementary Table 2. Secondary outcomes from Trial 1: Achievement of QOF indicators relating to the implementation packages; and non-trial related QOF indicators. All adjusted for covariates and baseline achievement of primary outcomes.**

|  | Unadjusted model estimates | | | | Adjusted model estimates | | | |
| --- | --- | --- | --- | --- | --- | --- | --- | --- |
|  | Diabetes control (n=40; mean) | Risky prescribing (n=40; mean) | Difference (97.5% CI) | p-value | Diabetes control (n=40; mean) | Risky prescribing (n=40; mean) | Difference (97.5% CI) | p-value |
| Indicator (QOF code, 2015-2016) | | | | | | | | |
| *Indicators relating to the diabetes implementation package* | | | | | | | | |
| The percentage of patients with diabetes, on the register, in whom the last blood pressure reading (measured in the preceding 12 months) is 150/90 mmHg or less (DM002) | 91.5 | 91.3 | 0.213 (-2.301, 2.727) | 0.847 | 91.3 | 91.2 | 0.111 (-2.391, 2.613) | 0.919 |
| The percentage of patients with diabetes, on the register, in whom the last blood pressure reading (measured in the preceding 12 months) is 140/80 mmHg or less (DM003) | 77.2 | 76.7 | 0.513 (-4.815, 5.841) | 0.826 | 76.4 | 76.3 | 0.063 (-5.246, 5.372) | 0.978 |
| The percentage of patients with diabetes, on the register, whose last measured total serum cholesterol (measured in the preceding 12 months) is 5 mmol/mol or less (DM004) | 79.5 | 79.3 | 0.242 (-3.553, 4.038) | 0.884 | 79.0 | 79.2 | -0.121 (-3.972, 3.730) | 0.943 |
| The percentage of patients with diabetes, on the register, with a diagnosis of nephropathy (clinical proteinuria) or micro-albuminuria who are currently treated with an ACE-I (or ARBs) (DM006)^*^ | 94.4 (6.6) | 89.9 (8.1) | - | - | - | - | - | - |
| The percentage of patients with diabetes, on the register, in whom the last IFCC-HbA1c is 59 mmol/mol or less in the preceding 12 months (DM007) | 69.9 | 68.1 | 1.871 (-3.037, 6.778) | 0.386 | 69.5 | 68.3 | 1.272 (-3.482, 6.025) | 0.541 |
| The percentage of patients with diabetes, on the register, in whom the last IFCC-HbA1c is 64 mmol/mol or less in the preceding 12 months (DM008) | 78.0 | 76.7 | 1.326 (-3.156, 5.809) | 0.501 | 77.5 | 76.8 | 0.706 (-3.533, 4.944) | 0.704 |
| The percentage of patients with diabetes, on the register, in whom the last IFCC-HbA1c is 75 mmol/mol or less in the preceding 12 months (DM009) | 88.1 | 87.2 | 0.830 (-2.968, 4.629) | 0.619 | 87.7 | 87.4 | 0.323 (-3.261, 3.907) | 0.837 |
| The percentage of patients with diabetes, on the register, with a record of a foot examination and risk classification within the preceding 12 months (DM012) | 85.9 | 83.5 | 2.374 (-2.989, 7.737) | 0.315 | 86.8 | 84.6 | 2.195 (-2.598, 6.988) | 0.297 |
| The percentage of patients newly diagnosed with diabetes, on the register, in the preceding 1 April to 31 March who have a record of being referred to a structured education programme within 9 months after entry on to the diabetes register (DM014)^*^ | 92.3 (18.9) | 91.8 (14.3) | - | - | - | - | - | - |
| The percentage of patients with diabetes, on the register, who have had influenza immunisation in the preceding 1 August to 31 March (DM018)^*^ | 95.8 (5.0) | 96.3 (3.3) | - | - | - | - | - | - |
|  | Diabetes control (n=40; mean) | Risky prescribing (n=40; mean) | Difference (97.5% CI) | p-value | Diabetes control (n=40; mean) | Risky prescribing (n=40; mean) | Difference (97.5% CI) | p-value |
| *Non-trial related indicators* | | | | | | | | |
| The percentage of patients with asthma, on the register, who have had an asthma review in the preceding 12 months that includes an assessment of asthma control using the 3 RCP questions (AST003) | 77.4 | 76.2 | -1.227 (-5.153, 2.698) | 0.477 | 77.5 | 76.4 | -1.077 (-5.244, 3.089) | 0.555 |
| The percentage of patients with coronary heart disease with a record in the preceding 12 months that aspirin, an alternative anti-platelet therapy, or an anti-coagulant is being taken (CHD005) | 96.1 (4.1) | 94.9 (3.5) | - | - | - | - | - | - |
| The percentage of patients with coronary heart disease who have had influenza immunisation in the preceding 1 August to 31 March (CHD007) | 95.7 (6.8) | 96.8 (3.7) | - | - | - | - | - | - |
| The percentage of patients with schizophrenia, bipolar affective disorder and other psychoses who have a comprehensive care plan documented in the record (in the preceding 12 months) agreed between individuals, their family and/or carers as appropriate (MH002) | 93.9 (5.4) | 87.1 (18.1) | - | - | - | - | - | - |
| The percentage of patients with schizophrenia, bipolar affective disorder and other psychoses who have a record of blood pressure in the preceding 12 months (MH003) | 92.1 (7.8) | 92.7 (5.9) | - | - | - | - | - | - |
| The percentage of patients with any or any combination of the following conditions: CHD, PAD, stroke or TIA, hypertension, diabetes, COPD, CKD, asthma, schizophrenia, bipolar affective disorder or other psychoses whose notes record smoking status in the preceding 12 months (SMOK002) | 95.5 | 96.5 | 0.924 (-0.465, 2.313) | 0.133 | 95.5 | 96.5 | 1.009 (-0.360, 2.377) | 0.095 |
| The percentage of patients aged 15 or over who are recorded as current smokers who have a record of an offer of support and treatment within the preceding 24 months (SMOK004) | 90.7 (9.8) | 90.1 (8.3) | - | - | - | - | - | - |
| The percentage of patients with any or any combination of the following conditions: CHD, PAD, stroke or TIA, hypertension, diabetes, COPD, CKD, asthma, schizophrenia, bipolar affective disorder or other psychoses who are recorded as current smokers who have a record of an offer of support and treatment within the preceding 12 months (SMOK005) | 97.4 (3.2) | 97.2 (4.1) | - | - | - | - | - | - |

^*^ Formal statistical testing was inappropriate due to violation of the modelling assumptions for the following trial-related indicators DM006, DM014, DM018; and the following non-trial related indicators: CHD005, CHD007, MH002, MH003, SMOK004, and SMOK005. Summary statistics only are presented for these indicators.

^†^the HbA1c and total serum cholesterol continuous intermediate clinical outcomes were analysed using a log transformation in order to satisfy the modelling assumptions. The predicted means presented are on the untransformed (original) scale but the estimated intervention effect (and 97.5% CI) are on the log scale.

* if urine albumin:creatinine ratio ≥3, or retinopathy, or record of cerebrovascular accident or transient ischemic attack;

QOF = quality and outcomes framework; CI = confidence interval; ACE-I = angiotensin-converting-enzyme inhibitor; ARB = angiotensin receptor blocker; IFCC = International Federation of Clinical Chemistry and Laboratory Medicine; HbA1c = haemoglobin A1c; RCP = Royal College of Physicians; CHD = coronary heart disease; PAD = peripheral arterial disease; TIA = transient ischemic attack; COPD = chronic obstructive pulmonary disease; CKD = chronic kidney disease (stage 3-5); Variables controlled for in the adjusted analyses were: practice-level baseline list size, CCG, pre-intervention achievement against primary outcomes, and total OQF score 2014-15.
