# Supplementary material for: An adaptable implementation package targeting evidence-based indicators in primary care: A pragmatic cluster-randomised evaluation
Source: PLoS Med. 2020 Feb 28;17(2):e1003045. doi: 10.1371/journal.pmed.1003045 (PMC7048270; doi:10.1371/journal.pmed.1003045)
Supplement: S3 Table — All adjusted for covariates and baseline achievement of primary outcomes. Values are percentage achievement, unless otherwise stated. Variables controlled for in the adjusted analyses were as follows: patient-level sex and age, and practice-level baseline list size, CCG, pre-intervention achievement against primary outcomes, total QOF score 2014–2015, and proportion of patients with 0–3 comorbidities. *If urine albumin:creatinine ratio ≥3, or retinopathy, or record of cerebrovascular accident or transient ischemic attack. AF, atrial fibrillation; BP, blood pressure; CCG, clinical commissioning group; CHA2DS2-VASc, congestive heart failure, hypertension, age>75, diabetes, stroke, vascular disease, age between 65 and 74, and female sex; CHD, coronary heart disease; CI, confidence interval; CKD, chronic kidney disease; CVD, cardiovascular disease; HTN, hypertension; PAD, peripheral arterial disease; QOF, Quality Outcomes Framework; TIA, transient ischemic attack. (DOCX) [file pmed.1003045.s003.docx]

**Supplementary Table 3. Secondary outcomes from Trial 2: Achievement of individual indicators that contributed to composite outcomes; processes of care; continuous intermediate clinical outcomes. All adjusted for covariates and baseline achievement of primary outcomes. Values are % achievement, unless otherwise stated.**

|  | Unadjusted model estimates | | | | Adjusted model estimates | | | |
| --- | --- | --- | --- | --- | --- | --- | --- | --- |
|  | Blood pressure control (n=32; %) | Anticoagulation (n=32; %) | Odds ratio (97.5% CI) | p-value | Blood pressure control (n=32; %) | Anticoagulation (n=32; %) | Odds ratio (97.5% CI) | p-value |
| *Blood pressure control indicators that contributed to the composite outcome*  (indicators refer to preceding 12 months; all patients aged <80y unless stated) | | | | | | | | |
| HTN and last recorded BP <140/90 mmHg | 54.5 | 52.4 | 1.087 (0.922, 1.280) | 0.256 | 54.7 | 52.7 | 1.082 (0.949, 1.233) | 0.179 |
| HTN, aged ≥80y and last recorded BP <150/90 mmHg | 78.8 | 77.7 | 1.063 (0.867, 1.305) | 0.500 | 78.9 | 77.8 | 1.065 (0.892, 1.270) | 0.427 |
| Type 2 diabetes and last recorded BP <140/80 mmHg, or <130/80 if complications* | 45.1 | 44.9 | 1.010 (0.832, 1.226) | 0.908 | 44.5 | 45.0 | 0.982 (0.809, 1.191) | 0.830 |
| CKD and proteinuria and last recorded BP <130/80 mmHg | 28.8 | 26.8 | 1.102 (0.881, 1.379) | 0.329 | 27.5 | 26.9 | 1.034 (0.803, 1.331) | 0.767 |
| CHD and last recorded BP <140/90 mmHg | 69.6 | 68.1 | 1.074 (0.892, 1.294) | 0.390 | 69.3 | 67.9 | 1.070 (0.923, 1.242) | 0.304 |
| PAD and last recorded BP <140/90 mmHg | 63.5 | 59.8 | 1.169 (0.915, 1.493) | 0.154 | 64.3 | 59.7 | 1.213 (0.982, 1.498) | 0.040 |
| Stroke/TIA and last recorded BP <140/90 mmHg | 62.4 | 62.1 | 1.014 (0.838, 1.227) | 0.867 | 62.6 | 62.3 | 1.011 (0.867, 1.180) | 0.868 |
| CVD risk of ≥20% and last recorded BP <140/90 mmHg | 49.7 | 47.4 | 1.096 (0.942, 1.274) | 0.174 | 50.3 | 47.9 | 1.100 (0.967, 1.252) | 0.099 |
|  | Blood pressure control (n=32; mean | Anticoagulation (n=32; mean | Difference (97.5% CI) | p-value | Blood pressure control (n=32; mean | Anticoagulation (n=32; mean | Difference (97.5% CI) | p-value |
| *Continuous intermediate clinical outcomes*  Last recorded value in previous 12 months | | | | | | | | |
| Systolic blood pressure (mmHg) | 134.2 | 133.9 | 0.270 (-1.000, 1.540) | 0.634 | 134.3 | 134.0 | 0.274 (-0.926, 1.474) | 0.608 |
| Diastolic blood pressure (mmHg) | 76.9 | 77.1 | -0.225 (-1.193, 0.744) | 0.603 | 76.8 | 77.0 | -0.203 (-1.230, 0.825) | 0.658 |
|  | Blood pressure control (n=32; %) | Anticoagulation (n=32; %) | Odds ratio (97.5% CI) | p-value | Blood pressure control (n=32; %) | Anticoagulation (n=32; %) | Odds ratio (97.5% CI) | p-value |
| *Process measures* | | | | | | | | |
| The proportion of eligible patients with measured blood pressure in the previous 12 months | 87.6 | 88.5 | 0.918 (0.665, 1.267) | 0.551 | 88.7 | 89.6 | 0.912 (0.634, 1.311) | 0.568 |
| *Anticoagulation in atrial fibrillation indicators that contributed to the composite outcome* | | | | | | | | |
| On AF register, male, CHA_2_DS_2_-VASc score = 1 and current prescription of anticoagulation | 64.5 | 56.2 | 0.705 (0.446, 1.114) | 0.086 | 65.2 | 55.7 | 0.672 (0.369, 1.223) | 0.136 |
| On AF register, male, CHA_2_DS_2_-VASc = 1 and current prescription of anticoagulation or contraindication for anticoagulation | 67.6 | 59.3 | 0.696 (0.432, 1.124) | 0.090 | 68.5 | 59.8 | 0.683 (0.366, 1.275) | 0.170 |
| On AF register and CHA_2_DS_2_-VASc ≥2 and current prescription for anticoagulation | 76.3 | 74.5 | 0.909 (0.709, 1.165) | 0.389 | 76.4 | 75.5 | 0.954 (0.787, 1.157) | 0.588 |
| On AF register and CHA_2_DS_2_-VASc ≥2 and current prescription for anticoagulation or contraindication for anticoagulation | 81.9 | 80.3 | 0.900 (0.681, 1.190) | 0.399 | 82.7 | 80.8 | 0.880 (0.692, 1.119) | 0.234 |

CI = confidence interval; HTN = hypertension; BP = blood pressure; * if urine albumin:creatinine ratio ≥3, or retinopathy, or record of cerebrovascular accident or transient ischemic attack; CKD = chronic kidney disease; CHD = coronary heart disease; PAD = peripheral arterial disease; TIA = transient ischemic attack; CVD = cardiovascular disease; AF = atrial fibrillation; CHA_2_DS_2_-VASc = congestive heart failure, hypertension, age>75, diabetes mellitus, stroke, and vascular disease

Variables controlled for in the adjusted analyses were: patient-level sex and age, and practice-level baseline list size, CCG, pre-intervention achievement against primary outcomes, total QOF score 2014-15, and proportion of patients with 0-3 comorbidities
