# Supplementary material for: An adaptable implementation package targeting evidence-based indicators in primary care: A pragmatic cluster-randomised evaluation
Source: PLoS Med. 2020 Feb 28;17(2):e1003045. doi: 10.1371/journal.pmed.1003045 (PMC7048270; doi:10.1371/journal.pmed.1003045)
Supplement: S4 Table — All adjusted for covariates and baseline achievement of primary outcomes. Note: Formal statistical testing was inappropriate due to violation of the modelling assumptions for the following trial-related indicators: AF006, AF007; and the following non-trial related indicators: CHD005, CHD007, MH002, MH003, SMOK004, and SMOK005. Summary statistics only are presented for these indicators. Variables controlled for in the adjusted analyses were as follows: practice-level baseline list size, CCG, pre-intervention achievement against primary outcomes and total QOF score 2014–2015. CCG, clinical commissioning group; CHA2DS2-VASc, congestive heart failure, hypertension, age>75, diabetes, stroke, vascular disease, age between 65 and 74, and female sex; CHD, coronary heart disease; CI, confidence interval; CKD, chronic kidney disease; COPD, chronic obstructive pulmonary disease; PAD, peripheral arterial disease; QOF, Quality and outcomes framework; RCP, Royal College of Physicians; TIA, transient ischemic attack. (DOCX) [file pmed.1003045.s004.docx]

**Supplementary Table 4: Secondary outcomes from Trial 2: Achievement of QOF indicators relating to the implementation packages; and non-trial-related QOF indicators. All adjusted for covariates and baseline achievement of primary outcomes.**

|  | Unadjusted model estimates | | | | Adjusted model estimates | | | |
| --- | --- | --- | --- | --- | --- | --- | --- | --- |
|  | Blood pressure control (n=32; mean) | Anticoagulation (n=32; mean) | Difference (97.5% CI) | p-value | Blood pressure control (n=32; mean) | Anticoagulation (n=32; mean) | Difference (97.5% CI) | p-value |
| Indicator (QOF code, 2015-2016) | | | | | | | | |
| *Indicators relating to the blood pressure implementation package* | | | | | | | | |
| The percentage of patients with coronary heart disease in whom the last blood pressure reading (measured in the preceding 12 months) is 150/90 mmHg or less (CHD002) | 90.7 | 91.6 | -0.948 (-3.612, 1.717) | 0.417 | 90.8 | 91.7 | -0.894 (-3.125, 1.338) | 0.359 |
| The percentage of patients with hypertension in whom the last blood pressure reading (measured in the preceding 12 months) is 150/90 mmHg or less (HYP006) | 83.0 | 81.7 | 1.349 (-1.488, 4.186) | 0.279 | 83.0 | 82.1 | 0.887 (-1.616, 3.391) | 0.416 |
| The percentage of patients with a history of stroke or transient ischemic attack in whom the last blood pressure reading (measured in the preceding 12 months) is 150/90 mmHg or less (STIA003) | 87.8 | 87.7 | 0.029 (-3.357, 3.415) | 0.984 | 87.6 | 87.8 | -0.169 (-3.624, 3.286) | 0.910 |
|  | Blood pressure control (n=32; mean | Anticoagulation (n=32; mean) | Difference (97.5% CI) | p-value | Blood pressure control (n=32; mean) | Anticoagulation (n=32; mean) | Difference (97.5% CI) | p-value |
| *Indicators relating to the anticoagulation in atrial fibrillation implementation package* | | | | | | | | |
| The percentage of patients with atrial fibrillation in whom stroke risk has been assessed using the CHA_2_DS_2_-VASc score risk stratification scoring system in the preceding 3 years (excluding those patients with a previous CHADS2 or CHA_2_DS_2_-VASc score of 2 or more) (AF006)^*^ | 96.9 (3.4) | 97.1 (3.9) | - | - | - | - | - | - |
| In those patients with atrial fibrillation with a record of CHA_2_DS_2_-VASc score of 2 or more, the percentage of patients who are currently treated with anticoagulation drug therapy (AF007)^*^ | 86.2 (6.9) | 87.3 (9.9) | - | - | - | - | - | - |
|  | Blood pressure control (n=32; mean | Anticoagulation (n=32; mean) | Difference (97.5% CI) | p-value | Blood pressure control (n=32; mean) | Anticoagulation (n=32; mean) | Difference (97.5% CI) | p-value |
| *Non-trial related indicators* | | | | | | | | |
| The percentage of patients with asthma, on the register, who have had an asthma review in the preceding 12 months that includes an assessment of asthma control using the 3 RCP questions (AST003) | 77.0 | 77.5 | 0.421 (-4.475, 5.318) | 0.844 | 77.5 | 77.2 | -0.234 (-4.721, 4.253) | 0.904 |
| The percentage of patients with coronary heart disease with a record in the preceding 12 months that aspirin, an alternative anti-platelet therapy, or an anti-coagulant is being taken (CHD005) | 93.7 (4.8) | 95.1 (4.0) | - | - | - | - | - | - |
| The percentage of patients with coronary heart disease who have had influenza immunisation in the preceding 1 August to 31 March (CHD007) | 94.3 (5.8) | 96.7 (3.9) | - | - | - | - | - | - |
| The percentage of patients with schizophrenia, bipolar affective disorder and other psychoses who have a comprehensive care plan documented in the record (in the preceding 12 months) agreed between individuals, their family and/or carers as appropriate (MH002) | 87.5 (16.7) | 89.9 (14.8) | - | - | - | - | - | - |
| The percentage of patients with schizophrenia, bipolar affective disorder and other psychoses who have a record of blood pressure in the preceding 12 months (MH003) | 90.0 (9.6) | 89.8 (7.3) | - | - | - | - | - | - |
| The percentage of patients with any or any combination of the following conditions: CHD, PAD, stroke or TIA, hypertension, diabetes, COPD, CKD, asthma, schizophrenia, bipolar affective disorder or other psychoses whose notes record smoking status in the preceding 12 months (SMOK002) | 95.4 | 95.7 | 0.290 (-1.326, 1.905) | 0.682 | 95.6 | 95.7 | 0.090 (-1.263, 1.443) | 0.878 |
| The percentage of patients aged 15 or over who are recorded as current smokers who have a record of an offer of support and treatment within the preceding 24 months (SMOK004) | 89.1 (9.3) | 89.5 (10.7) | - | - | - | - | - | - |
| The percentage of patients with any or any combination of the following conditions: CHD, PAD, stroke or TIA, hypertension, diabetes, COPD, CKD, asthma, schizophrenia, bipolar affective disorder or other psychoses who are recorded as current smokers who have a record of an offer of support and treatment within the preceding 12 months (SMOK005) | 97.4 (3.3) | 96.8 (4.7) | - | - | - | - | - | - |

^*^ Formal statistical testing was inappropriate due to violation of the modelling assumptions for the following trial-related indicators: AF006, AF007; and the following non-trial related indicators: CHD005, CHD007, MH002, MH003, SMOK004, and SMOK005. Summary statistics only are presented for these indicators.

QOF = Quality and outcomes framework; CI = confidence interval; CHA_2_DS_2_-VASc = congestive heart failure, hypertension, age>75, diabetes mellitus, stroke, and vascular disease; RCP = Royal College of Physicians; CHD = coronary heart disease; PAD = peripheral arterial disease; TIA = transient ischemic attack; COPD = chronic obstructive pulmonary disease; CKD = chronic kidney disease;

Variables controlled for in the adjusted analyses were: practice-level baseline list size, CCG, pre-intervention achievement against primary outcomes and total QOF score 2014-15
