# Supplementary material for: An adaptable implementation package targeting evidence-based indicators in primary care: A pragmatic cluster-randomised evaluation
Source: PLoS Med. 2020 Feb 28;17(2):e1003045. doi: 10.1371/journal.pmed.1003045 (PMC7048270; doi:10.1371/journal.pmed.1003045)
Supplement: S5 Table — Data from an earlier work package of the ASPIRE programme were used to inform the trial sample size assumptions. Mean cluster size, cluster size coefficient of variation, ICC, and mean achievement rates were calculated using real data from practices within West Yorkshire for each primary outcome indicator. aMean achievement is the control arm achievement rate for each primary outcome indicator estimated using data available from the earlier work package. ICC, intra-cluster correlation coefficient. (DOCX) [file pmed.1003045.s005.docx]

**Supplementary Table 5. Key sample size assumptions**

|  | Diabetes Control | Risky prescribing | Blood pressure control | Anticoagulation in atrial fibrillation |
| --- | --- | --- | --- | --- |
| Mean cluster size (number of eligible patients per practice) | 280 | 420 | 800 | 55 |
| Coefficient of variation of cluster size | 0.60 | 0.65 | 0.67 | 0.79 |
| Intra-cluster correlation coefficient (ICC) | 0.06 | 0.03 | 0.06 | 0.06 |
| Mean achievement^a^ | 43.0% | 89.0% | 72.0% | 60.0% |

Data from an earlier work package of the ASPIRE programme was used to inform the trial sample size assumptions. Mean cluster size, cluster size coefficient of variation, intra-cluster correlation coefficient and mean achievement rates were calculated using real data from practices within West Yorkshire for each primary outcome indicator.

^a^ Mean achievement is the control arm achievement rate for each primary outcome indicator estimated using data available from the earlier work package.
