# Supplementary material for: An adaptable implementation package targeting evidence-based indicators in primary care: A pragmatic cluster-randomised evaluation
Source: PLoS Med. 2020 Feb 28;17(2):e1003045. doi: 10.1371/journal.pmed.1003045 (PMC7048270; doi:10.1371/journal.pmed.1003045)
Supplement: S6 Table — aOne practice in the risky prescribing arm merged with a non-ASPIRE practice in advance of the final feedback report. bOne practice in the BP arm closed in advance of the third feedback report. cOnly practices receiving an initial outreach visit were offered additional support; these practices are used as the denominator in the percentages presented. dThese granted access to the computerised searches (all arms) and prompts (risky prescribing only). BP, blood pressure. (DOCX) [file pmed.1003045.s006.docx]

**Supplementary Table 6. Intervention delivery across trial practices**

|  | Diabetes control  (n=40) | Risky prescribing (n=40) | Blood pressure control  (n=32) | Anticoagulation in atrial fibrillation  (n=32) | Total  (n=144) |
| --- | --- | --- | --- | --- | --- |
| Audit reports delivered quarterly | 40 (100%) | 39^a^ (98%) | 31^b^ (97%) | 32 (100%) | 142 (99%) |
| *Outreach visits and additional support* | | | | | |
| Initial outreach visit delivered | 20 (50%) | 25 (63%) | 11 (34%) | 11 (34%) | 67 (47%) |
| Received additional support^c^ | 5 (25%) | 6 (24%) | 2 (18%) | 3 (27%) | 16 (24%) |
| Second outreach visit delivered | 2 (5%) | 3 (8%) | 1 (3%) | 2 (6%) | 8 (6%) |
| *Organisational groups* | | | | | |
| Joined organisational groups^d^ | 38 (95%) | 40 (100%) | 23 (72%) | 25 (78%) | 126 (88%) |

^a^ One practice in the risky prescribing arm merged with a non-ASPIRE practice in advance of the final feedback report; ^b^ One practice in the blood pressure arm closed in advance of the third feedback report; ^c^ Only practices receiving an initial outreach visit were offered additional support; these practices are used as the denominator in the percentages presented; ^d^ These granted access to the computerised searches (all arms) and prompts (risky prescribing only)
