# Supplementary figures and images for: An adaptable implementation package targeting evidence-based indicators in primary care: A pragmatic cluster-randomised evaluation
Source: PLoS Med. 2020 Feb 28;17(2):e1003045. doi: 10.1371/journal.pmed.1003045 (PMC7048270; doi:10.1371/journal.pmed.1003045)

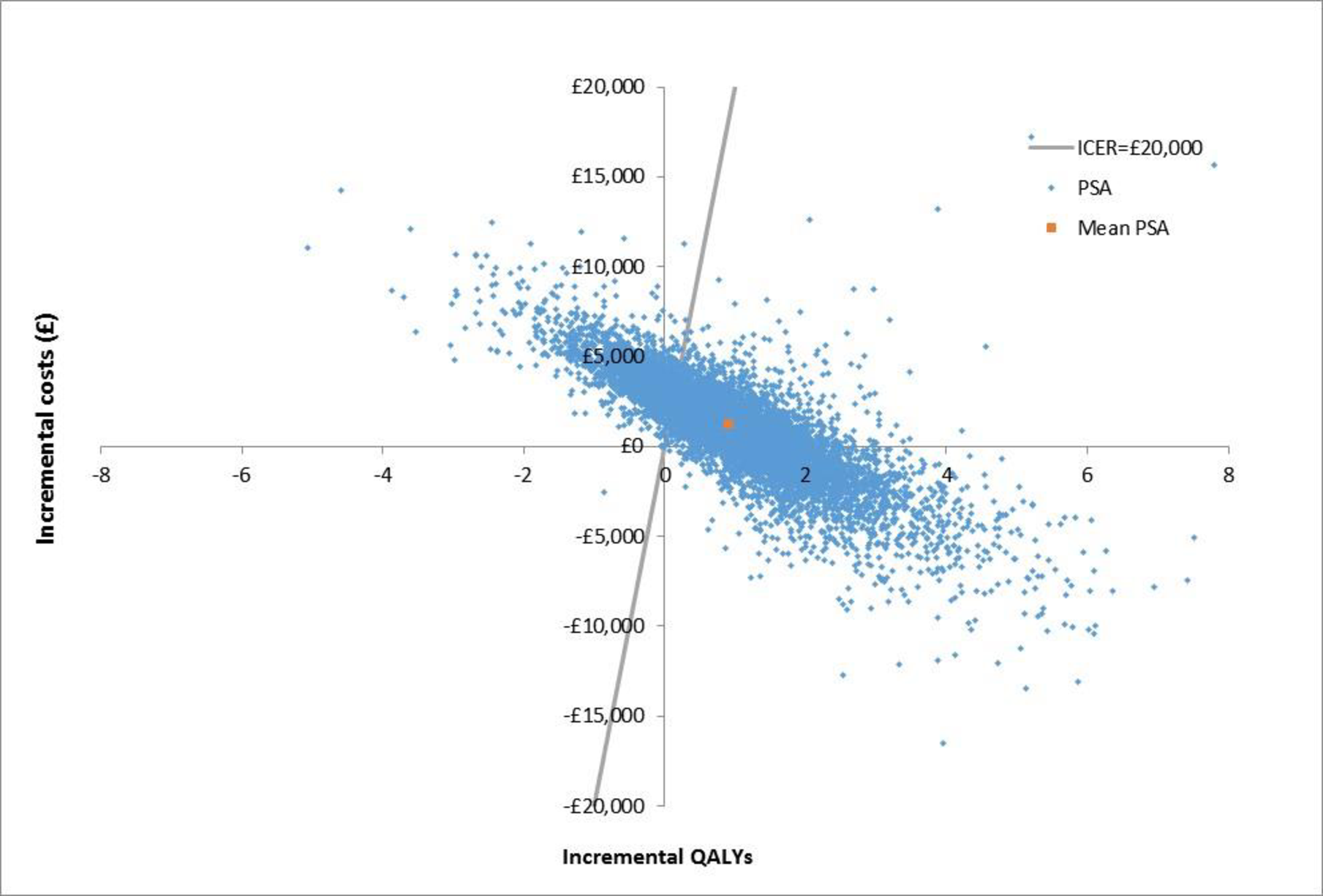

Supplement: S1 Fig — ICER, incremental cost-effectiveness ratio; PSA, probability sensitivity analysis; QALY, quality-adjusted life year. (TIF) [file pmed.1003045.s007.tif]

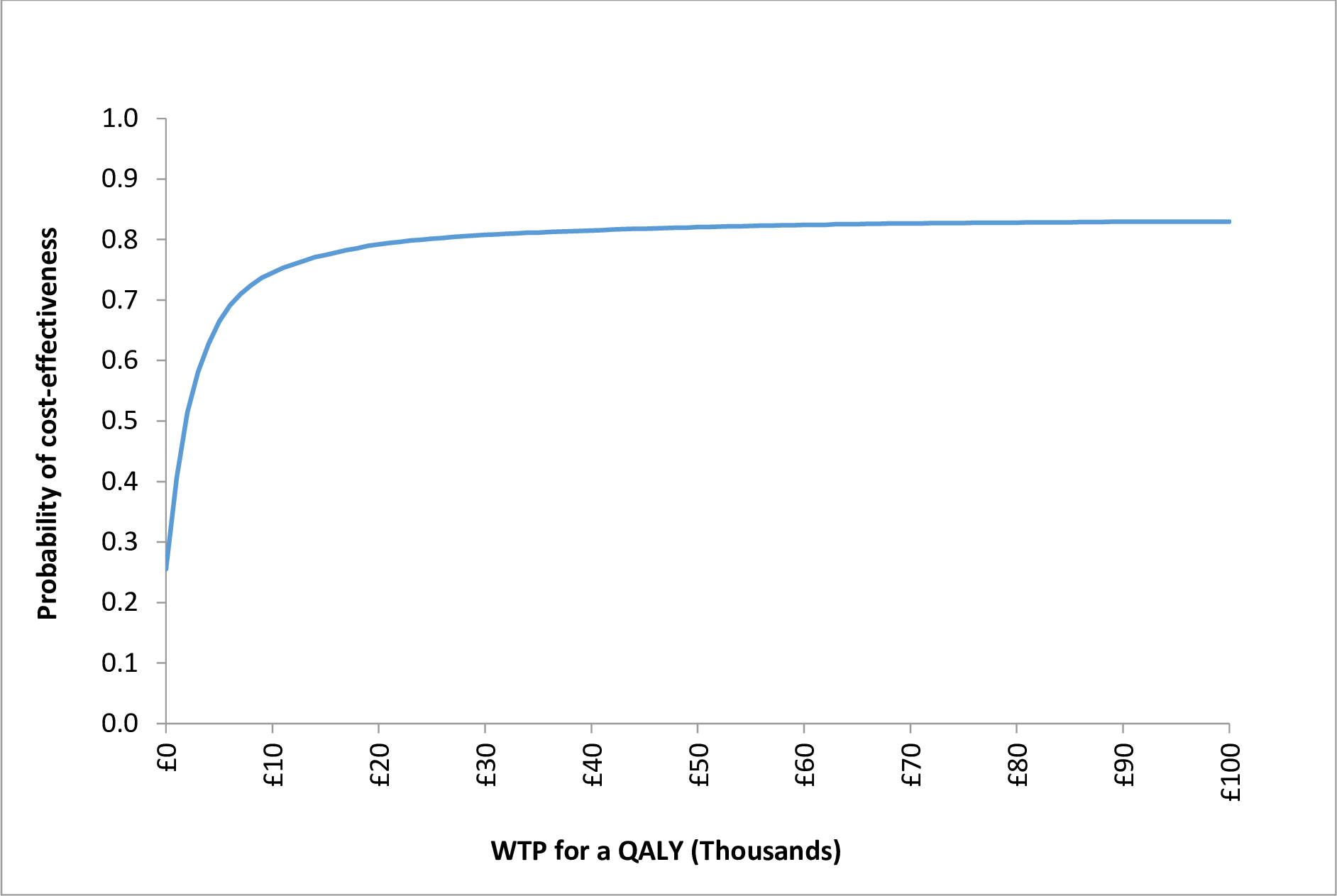

Supplement: S2 Fig — QALY, quality-adjusted life year; WTP, Willingness to Pay. (TIF) [file pmed.1003045.s008.tif]

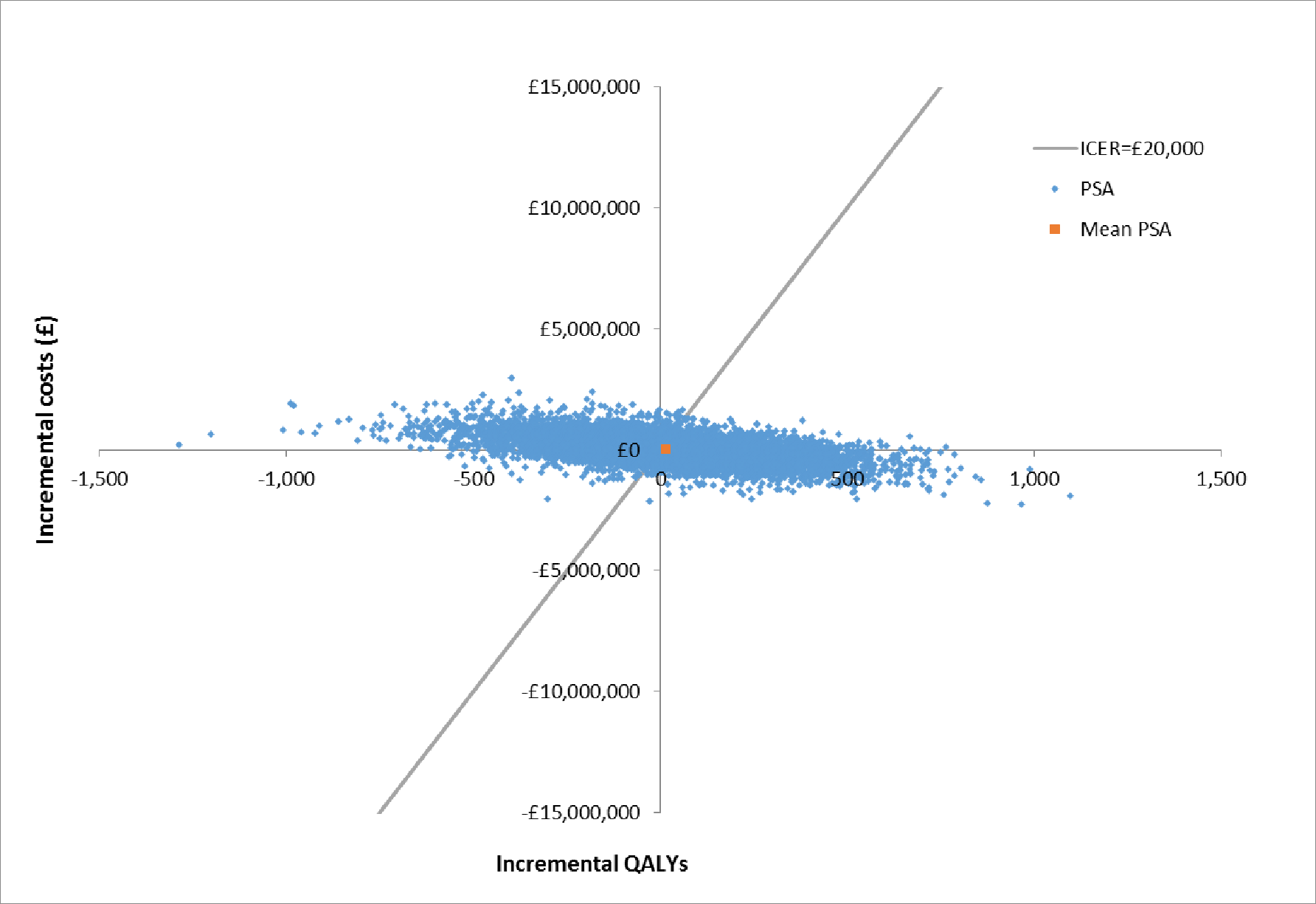

Supplement: S3 Fig — BP, blood pressure; ICER, incremental cost-effectiveness ratio; PSA, probability sensitivity analysis; QALY, quality-adjusted life year. (TIF) [file pmed.1003045.s009.tif]

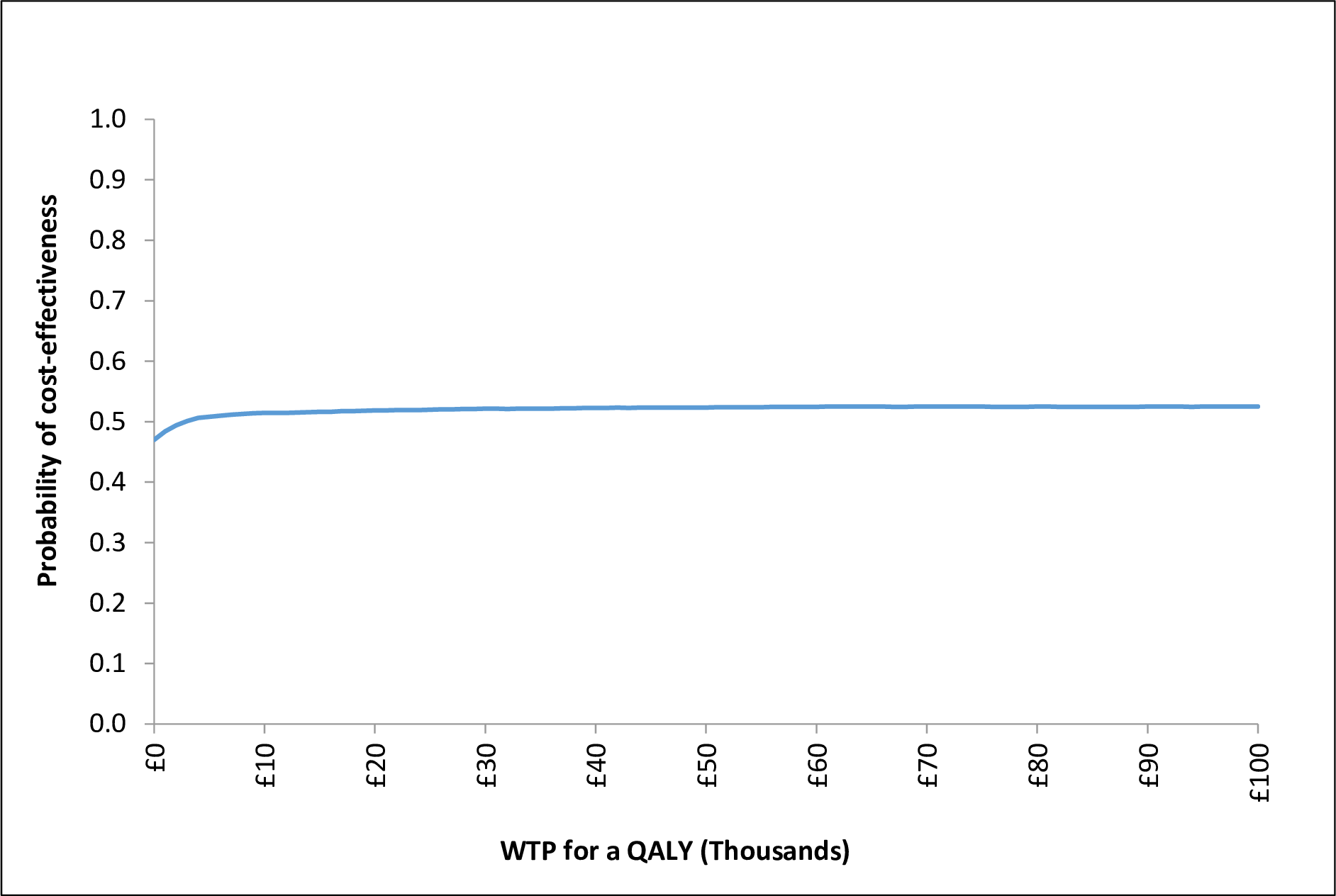

Supplement: S4 Fig — BP, blood pressure; QALY, quality-adjusted life year; WTP, Willingness to Pay. (TIF) [file pmed.1003045.s010.tif]
