# Supplementary material for: An adaptable implementation package targeting evidence-based indicators in primary care: A pragmatic cluster-randomised evaluation
Source: PLoS Med. 2020 Feb 28;17(2):e1003045. doi: 10.1371/journal.pmed.1003045 (PMC7048270; doi:10.1371/journal.pmed.1003045)
Supplement: S1 Statistical Analysis Plan — (DOCX) [file pmed.1003045.s012.docx]

Supplementary Document 1. Pre-specified Statistical Analysis Plan

**Clinical Trials Research Unit**

**(CTRU)**

**University of Leeds**

**Final**

**Statistical Analysis Plan**

**Action to Support Practices Implementing** **Research Evidence (ASPIRE)**

**Version 2.0**

**March 2017**

Trial Statistician: Peter Heudtlass / Mike Holland

Supervising Statistician: Michelle Collinson

CTRU Scientific Lead: Professor Amanda Farrin

Senior Trial Co-ordinator / Manager: Lorna Barnard

CTRU Project Delivery Lead: Suzanne Hartley

Chief Investigator: Professor Robbie Foy

**Table of Contents**

[1. Introduction 3](#_Toc477255036)

[1.1 Background: The ASPIRE research programme 3](#_Toc477255037)

[1.2 Design 4](#_Toc477255038)

[1.3 Aims 6](#_Toc477255039)

[1.3.1 Primary Objective 6](#_Toc477255040)

[1.3.2 Secondary Objectives 6](#_Toc477255041)

[1.4 Sample size 6](#_Toc477255042)

[1.5 Planned analyses 7](#_Toc477255043)

[2. Endpoints 7](#_Toc477255044)

[2.1 Primary and secondary endpoints (by clinical topic) 7](#_Toc477255045)

[2.2 Other Secondary endpoints 9](#_Toc477255046)

[2.2.1 Quality and Outcome Framework (QOF) indicators 9](#_Toc477255047)

[2.2.2 Other analyses 14](#_Toc477255048)

[2.3 Derivation of endpoints and other data 15](#_Toc477255049)

[2.4 Missing data 15](#_Toc477255050)

[2.5 Non-standard circumstances 17](#_Toc477255051)

[3. Populations 17](#_Toc477255052)

[3.1 Eligibility 17](#_Toc477255053)

[3.2 Intention to treat population 17](#_Toc477255054)

[3.3 Screening population 17](#_Toc477255055)

[3.4 Sensitivity analysis population 17](#_Toc477255056)

[4. Data Handling 17](#_Toc477255057)

[4.1 Data monitoring 17](#_Toc477255058)

[4.2 Data validation 17](#_Toc477255059)

[4.2.1 CSU Data 17](#_Toc477255060)

[4.2.2 Outreach Visit Data 18](#_Toc477255061)

[4.2.3 Other data 18](#_Toc477255062)

[5. Data Analysis 18](#_Toc477255063)

[5.1 General calculations 18](#_Toc477255064)

[5.2 Study summary 18](#_Toc477255065)

[5.2.1 Milestones 19](#_Toc477255066)

[5.2.2 Screening 19](#_Toc477255067)

[5.2.3 Practice withdrawals / mergers / closures 19](#_Toc477255068)

[5.2.4 Baseline characteristics 19](#_Toc477255069)

[5.3 Primary endpoint analysis 19](#_Toc477255070)

[5.4 Secondary endpoint analysis 19](#_Toc477255071)

[5.4.1 Intervention effects on separate indicators that make up the composite outcomes 19](#_Toc477255072)

[5.4.2 Intervention effects on recorded processes of care 19](#_Toc477255073)

[5.4.3 Intervention effects on continuous intermediate clinical outcomes 20](#_Toc477255074)

[5.4.4 Intervention effects on relevant QOF indicators 20](#_Toc477255075)

[5.4.5 Fidelity of intervention delivery 20](#_Toc477255076)

[5.4.6 Time-varying effects 21](#_Toc477255105)

[5.4.7 Exploring the Hawthorne effect 22](#_Toc477255106)

[5.4.8 Relationship between Care Quality Commission ratings and primary outcomes 22](#_Toc477255107)

[5.4.9 To explore the relationship between achievement and engagement for process evaluation practices 22](#_Toc477255108)

[5.5 Sensitivity analysis 23](#_Toc477255109)

[5.5.1 Missing data at patient level 23](#_Toc477255110)

[5.5.2 Missing data at practice level 23](#_Toc477255111)

[6. Reporting and Dissemination of the Results 23](#_Toc477255112)

[7. References 24](#_Toc477255113)

[Approval of Analysis Plan 25](#_Toc477255114)

# 1. Introduction

## Background: The ASPIRE research programme

The ASPIRE research programme comprises a series of five interlinked projects or work packages to develop and evaluate an intervention package which can be adapted to target implementation of high impact clinical practice recommendations in general practice:

- WP1a aimed to identify high impact clinical practice recommendations.
- WP1b aimed to measure and analyse levels of adherence to high impact clinical practice recommendation through cross sectional analysis of patient data to identify up to 8 high impact recommendations with greatest scope for improvement (low adherence) and explore variations in adherence.
- WP2a aimed to develop an adaptable intervention package to target the implementation of each recommendation through interviews to explore barriers to and enablers of adherence to each of the eight selected high impact recommendations in general practice; matching behaviour change techniques to identified barriers and enablers; development of an intervention package (computerised decision support, audit and feedback, and outreach educational visits) which can be adapted to target different types of recommendation.
- WP2b aims to evaluate the effects and cost-effectiveness of the adapted intervention package in targeting the implementation of high impact recommendations through intervention piloting and formative evaluation and two cluster randomised controlled trials of general practices to determine effects of the intervention package adapted for four high impact (or exemplar) recommendations and modelling of intervention cost-effectiveness.
- WP2c aims to conduct an in-depth, process evaluation to examine intervention delivery and mechanisms of action, as well as unintended consequences.

Our overall approach is consistent with Medical Research Council (MRC) guidance on the development and evaluation of complex interventions. [1] It will: lead to the development of an evidence-based and practical implementation field manual for general practice; and form the foundation for further research evaluating the effects of the adapted intervention package for other high impact recommendations.

This analysis plan relates to the two cluster randomised trials in WP2b and quantitative analysis of data from the process evaluation in WP2c. The qualitative analysis of WP2c is not covered in this analysis plan.

## 1.2 Design

The ASPIRE trials are two pragmatic multi-centre cluster randomised controlled trials with balanced incomplete block designs running in parallel [2, 3]. Each trial aims to evaluate the effect of using tailored intervention packages on adherence to two of four high impact clinical practice recommendations. The intervention packages have been developed and piloted in previous work packages. Figure 1 details the trial design.

Trial 1 will evaluate the intervention packages for the Diabetes and Risky Prescribing recommendations whilst Trial 2 will evaluate the intervention packages for the Hypertension and Atrial Fibrillation recommendations.

We recruited and randomised 178 General Practices across West Yorkshire. Practices were first randomised to one of the two trials or to a third group of control practices not receiving any intervention. Practices from the Bradford Districts CCG were only randomised to either trial 1 or to the third group not receiving any intervention, because of the presence of another dedicated Atrial Fibrillation intervention in that CCG. Secondly, practices within the two trials were randomised to the adapted intervention package for a single recommendation.

The required sample size for trial 1 was 80 practices and for trial 2, 64 practices. The difference between the total required sample size (80+64=144) and the actual total recruitment (178) determined the size of the group of control practices not receiving any intervention: 34 (178-144). We are gathering comparative data on these non-intervention practices but they will not receive any of the intervention packages. Within the two trials, each practice will act as the control for the other recommendation within the trial to which they are randomised.

Cluster randomisation has been chosen both because quality improvement initiatives target the staff within general practices and to reduce between-group contamination as the intervention aims to change clinical practice. The proposed balanced incomplete block design will be used to equalise Hawthorne effects whilst maximising power and efficiency [2, 3]. It will also reduce the risk of overburdening exposure to more than one intervention. The balanced incomplete block design requires all intervention packages within the trial to be independent in terms of their outcomes; any dependencies could dilute the intervention effect. To minimise this risk, we plan two 2-arm trials instead of one 4-arm trial.

To evaluate the level of adherence to each of the four recommendations we aim to analyse approximately 216,000 anonymised individual patient records (all patients who are targeted by at least one of the four recommendations) across the 144 sites (on average about 1,500 records per site) at 11 months post randomisation. The trial period was initially planned as 12 months from randomisation to final data download to cover a single financial year (this coincides with the one year reporting period of the Quality and Outcome Framework (QOF)). However, due to delays with the transfer of the baseline data (which was required for stratification), randomisation was delayed until late April 2015. The trial management group discussed the implications of this delay and decided not to postpone the final data download date. It was considered important to assess the trial outcomes at the end of a financial/QOF year as adherence to QOF-related NICE guidelines was assumed to have seasonal pattern. Therefore, the trial period was shortened from 12 to 11 months.

The aim of the trials is to determine whether appropriate care was provided – or targets achieved – in accordance with the recommendations.

Stage 1 (80:64:34)

Stage 2 (1:1 and 1:1)

No

Intervention

(all other

General Practices

**not** opting out)

N=34

Two stage randomisation

Stratification criteria:

- Baseline adherence to target recommendations (only at second randomisation stage)
- CCG (at both stages)
- Practice list size (at both stages)

Hypertension

N=32

Each practice receives an adapted intervention package tailored to the NICE recommendation for either Hypertension or Atrial Fibrillation and acts as a control for the other intervention in trial 2.

Trial 2

Atrial fibrillation

N=32

Risky prescribing

N=40

Follow-up at up to 11 months post randomisation.

Data will be sought from the CSU to inform primary and secondary endpoints.

Trial 1

Site identification:

General practices in West Yorkshire CCGs that use SystmOne will be eligible. Those that were involved in piloting the intervention packages or that will be involved in the process evaluation project will be excluded.

Analysis:

- Primary analysis
- Secondary analysis

Each practice receives an adapted intervention package tailored to the NICE recommendation for either Diabetes or Risky Prescribing and acts as a control for the other intervention in trial 1.

Data will be obtained at baseline to determine baseline characteristics and inform stratification.

NHS permission will be sought from the CCGs for eligible sites.

All eligible sites will go through the opt out process

Diabetes

N=40

Figure 1: Trial Flow Chart

## 1.3 Aims

We aim to evaluate the effectiveness of adapted implementation packages targeting four high impact clinical practice recommendations in general practice. The cost effectiveness analysis is the responsibility of the ASPIRE Health Economist and is described elsewhere.

### 1.3.1 Primary Objective

The primary objective is to assess the effectiveness of the implementation packages in promoting adherence to four targeted recommendations at 11 months post randomisation:

- Achievement of all recommended target levels of relevant outcome measures in patients with diabetes;
- Reduction of high-risk non-steroidal anti-inflammatory drug and antiplatelet prescribing;
- Achievement of recommended blood pressure targets for patients at high risk of cardiovascular events;
- Anticoagulation prescribing in eligible patients with atrial fibrillation.

### 1.3.2 Secondary Objectives

- To assess the intervention effects on separate indicators that make up the composite outcomes
- To assess the intervention effects on recorded processes of care
- To assess the intervention effects on continuous intermediate clinical outcomes (e.g. blood pressure)
- To assess the intervention effects on relevant Quality and Outcome Framework (QOF) indicators
- To report fidelity of intervention delivery overall and by recommendation, as per the TIDIER checklist
- To assess whether intervention fidelity is related to intervention effects
- To assess whether intervention effects vary over time using baseline and audit and feedback data
- To explore the Hawthorne effect using data from the non-intervention trials
- To explore the relationship between Care Quality Commission (CQC) ratings and the primary outcomes and to explore whether CQC ratings moderate intervention effect
- To explore the relationship between achievement and engagement for process evaluation practices

## 1.4 Sample size

The sample size calculations are based on data and analysis from Work Package 1b of the ASPIRE project. Particularly, we were able to estimate mean cluster size (number of targeted patients per practice by recommendation), coefficient of variation, intra-cluster correlation coefficient and control group achievement rates using real data from West Yorkshire practices (Table 1).

The median effect sizes on processes and outcomes of care for a range of single interventions in guideline implementation studies are around 4-9%.[2, 4] We intend to improve upon this by systematically targeting multiple enablers of and barriers to change. We will also be targeting recommendations with greater scope for improvement; lower baseline adherence was independently associated with greater effect size in a Cochrane Review of audit and feedback. [5] Hence, an estimated effect size of 15% for outcomes related to hypertension, diabetes and atrial fibrillation are realistic and clinically relevant. Control group achievement rates in risky prescribing are considerably higher (Table 1), and considering a ceiling effect, we consider 5% is a realistic and still clinically relevant effect size.

Depending on the recommendation, assumed ICCs range from 0.03 to 0.06, mean cluster sizes from 55 to 800 and the coefficient of variation of cluster sizes from 0.6 to 0.79 (Table 1). In order to achieve 90% power, and allowing for an alpha error rate of 2.5% (to adjust for two outcome comparisons) and after increasing the sample size by 10% to account for practice drop-out, we require 40 clusters per arm in trial 1 (diabetes and risky prescribing) and 32 clusters per arm in trial 2 (hypertension and atrial fibrillation).

Overall, we shall aim for 144 practices. We anticipate sufficient capacity from 330 practices in West Yorkshire. Having examined a range of cluster trials in primary care testing similar types of interventions (e.g. computerised prompts, audit and feedback); [6-9] numbers of participating practices ranged from 58 to 244. We therefore judge 144 practices to be feasible.

Table 1: Key sample size assumptions based on data from an earlier Work Package

|  | AF | Hypertension | Risky prescribing | Diabetes |
| --- | --- | --- | --- | --- |
| Mean number of patients per practice (cluster size) | 55 | 800 | 420 | 280 |
| Coefficient of variation (CV) of cluster size | 0.79 | 0.67 | 0.65 | 0.6 |
| Intra-cluster correlation coefficient (ICC) | 0.06 | 0.06 | 0.03 | 0.06 |
| Control group adherence | 0.6 | 0.72 | 0.89 | 0.43 |

## 1.5 Planned analyses

Data will be extracted from CSU 11 months after randomisation, during April 2016, and final analyses will be conducted. No interim or sub-group analyses are planned.

The statistical report of the final analysis will follow the guidance defined by the funder (http://www.journalslibrary.nihr.ac.uk/information-for-authors/programme-grants-for-applied-research).

# 2. Endpoints

## 2.1 Primary and secondary endpoints (by clinical topic)

The primary endpoint for each intervention package will be the level of adherence to a composite indicator representing the targeted recommendation at 11 months post randomisation. Secondary endpoints are the levels of adherence of the individual components of the composite indicators, recorded processes of care, and the intervention effects on continuous clinical outcomes at 11 months post randomisation. Primary and related secondary endpoints are described in detail in the following table.

**Table 2: Primary and secondary endpoints (by clinical topic)**

| Topic | Primary endpoints | Secondary endpoints |
| --- | --- | --- |
| Diabetes  (trial 1) | The proportion of patients with type 2 diabetes achieving all three of the following treatment targets (composite endpoint):  1) blood pressure below 140/80 mmHg (or 130/80 mmHg if there is kidney, eye or cerebrovascular damage);  2) HbA1c value below or equal to 59 mmol/mol and  3) total serum cholesterol level below or equal to 5.0 mmol/l. | 1. The proportion of patients with type 2 diabetes achieving each of the three treatment targets that make up the primary composite endpoint; 2. The proportion of patients with type 2 diabetes receiving each of the following nine processes of care in the previous 12 months:  (i) BP measured,  (ii) HbA1c levels measured,  (iii) full lipid profile performed,  (iv) micro-albuminuria testing performed,  (v) eGFR / serum creatinine testing performed,  (vi) foot care review,  (vii) eye screening,  (viii)BMI recorded,  (ix) smoking status recorded 3. The proportion of patients receiving all of the above processes of care (composite); 4. The proportion of patients with type 2 diabetes receiving all the processes of care as defined in 2) above with the exception of (vii) eye screening (composite of 8 processes of care); 5. Mean patient levels of: (i) systolic and (ii) diastolic blood pressure, (iii) HbA1c and (iv) total serum cholesterol (continuous intermediate clinical outcomes). |
| Risky prescribing  (trial 1) | The proportion of patients achieving at least one of the nine indicators of high-risk NSAID and anti-platelet prescribing (composite endpoint):  1) prescribing a traditional oral NSAID or low-dose aspirin in patients with a history of peptic ulceration without co-prescription of gastro-protection;  2) prescribing a traditional oral NSAID in patients aged 75 years or over without co-prescription of gastro-protection;  3) prescribing of a traditional oral NSAID and aspirin in patients aged 65 years or over without co-prescription of gastro-protection;  4) prescribing of aspirin and clopidogrel in patients aged 65 years or over without co-prescription of gastro-protection;  5) prescribing of warfarin and a traditional oral NSAID;  6) prescribing of warfarin and low-dose aspirin or clopidogrel without co-prescription of gastro-protection;  7) prescribing an oral NSAID in patients with heart failure;  8) prescribing an oral NSAID in patients prescribed both a diuretic and an angiotensin-converting-enzyme inhibitor (ACE-I) or angiotensin receptor blocker (ARB) and  9) prescribing an oral NSAID in patients with chronic kidney disease (CKD) | 1. The proportion of patients achieving each of the nine measures that make up the primary composite endpoint; 2. The proportion of patients achieving at least one of indicators 1-6 (gastro-intestinal composite); 3. The proportion of patients achieving at least one of indicators 8-9 (renal composite). |
| Hypertension  (trial 2) | The proportion of patients achieving at least one of the eight recommended targets for satisfactorily controlled blood pressure (composite endpoint):  1) BP <140/90 in patients aged under 80y with hypertension,  2) BP <150/90 in patients aged 80 years and over with hypertension,  3) BP < 140/80 in patients aged under 80 years with diabetes, and <130/80 if there are complications of diabetes,  4) BP <130/80 in patients aged under 80 years with chronic kidney disease and proteinuria,  5) BP <140/90 in patients aged under 80 years with coronary heart disease,  6) BP < 140/90 in patients aged under 80 years with peripheral arterial disease,  7) BP <140/90 in patients aged under 80 years with a history of stroke/TIA, and  8) BP <140/90 in patients aged under 80 years with a cardiovascular disease risk of 20% or higher.  NB Patients should only appear once, and where any appear in more than one group with differing BP targets, the lower target should take precedence. | 1. The proportion of patients achieving each of the 8 targets that make up the primary composite endpoint; 2. Mean patient levels of diastolic and systolic blood pressure (continuous intermediate clinical outcomes); 3. The proportion of patients with hypertension with measured BP in the previous 12 months (process of care). |
| Atrial Fibrillation  (trial 2) | The proportion of patients achieving at least one of the two indicators of atrial fibrillation (composite endpoint):  1) the proportion of men with atrial fibrillation and a CHA2DS2-VASc score of 1 prescribed anticoagulation therapy and  2) the proportion of all people with atrial fibrillation and a CHA2DS2-VASc score of 2 or above prescribed anticoagulation therapy. | 1. The proportion of patients achieving each of the 2 measures that make up the primary composite endpoint; 2. The proportion of patients achieving each of the two indicators of atrial fibrillation who also have contraindication for anti-coagulation:  (i) the proportion of men with atrial fibrillation and a CHA2DS2-VASc score of 1 prescribed anticoagulation therapy with contraindication for anti-coagulation and; (ii) the proportion of all people with atrial fibrillation and a CHA2DS2-VASc score of 2 or above prescribed anticoagulation therapy with contraindication for anti-coagulation; 3. The proportion of patients achieving at least one of the two indicators related to contraindication for anti-coagulation as defined in 2) above (composite).  This is termed ‘exception coding’ (i.e. clinician-judged exemption from treatment). |

## 2.2 Other Secondary endpoints

### 2.2.1 Quality and Outcome Framework (QOF) indicators

The Quality and Outcomes Framework is a performance management system whereby general practices are remunerated according to achievement of targets reflecting quality of care across four domains of clinical, organisational, patient experience and additional services [10]. Practice data collection for QOF operates on an annual cyclical basis from 1 April to 31 March. Several indicators map onto our trial recommendations (Table 3). We have also selected a series of non-trial-related indicators that represent different aspects of care and service delivery (Table 4). These will be used to measure unintended impacts (sentinel indicators) on quality of care and relate to four areas: coronary heart disease (an example of a long-term physical condition), mental health (long-term non-physical condition), smoking (health promotion) and asthma (long-term physical condition).

The endpoints of interest are described in detail in Tables 3 and 4.

**Table 3: Trial-related QOF Indicators 2015-16**

| **Domain** | **QOF indicator number** (2015–16) | **Indicator** | **Intended effect** | **Hypothesised plausible relationships** |
| --- | --- | --- | --- | --- |
| **Atrial fibrillation** | **AF006** | The percentage of patients with atrial fibrillation in whom stroke risk has been assessed using the CHA2DS2-VASc score risk stratification scoring system in the preceding 12 months (excluding those patients with a previous CHADS2 or CHA2DS2-VASc score of 2 or more). | Related to the denominator of the primary endpoints in trial 2, atrial fibrillation: “A composite of the proportion of men with atrial fibrillation and a CHA2DS2-VASc score of 1 prescribed anticoagulation therapy and the proportion of all people with atrial fibrillation and a CHA2DS2-VASc score of 2 or above prescribed anticoagulation therapy” |  |
| **Atrial fibrillation** | **AF007** | In those patients with atrial fibrillation with a record of a CHA2DS2-VASc score of 2 or more, the percentage of patients who are currently treated with anticoagulation drug therapy. | Identical to one of the components of the primary endpoint in trial 2, atrial fibrillation: “the proportion of all people with atrial fibrillation and a CHA2DS2-VASc score of 2 or above prescribed anticoagulation therapy” |  |
| **Secondary prevention of coronary heart disease** | **CHD002** | The percentage of patients with coronary heart disease in whom the last blood pressure reading (measured in the preceding 12 months) is 150/90 mmHg or less. | Related to one of the components of the primary endpoint in trial 2, hypertension: “The proportion of patients with satisfactorily controlled blood pressure according to recommended targets: […] BP <140/90 in patients aged under 80 years with coronary heart disease […]” | Potentially related to one of the components of the primary endpoint in trial 1, diabetes: “The proportion of patients with type 2 diabetes achieving […] BP below 140/80 mmHg (or 130/80 mmHg if there is kidney, eye or cerebrovascular damage)”. |
| **Hypertension** | **HYP006** | The percentage of patients with hypertension in whom the last blood pressure reading (measured in the preceding 12 months) is 150/90 mmHg or less. | Related to one of the components of the primary endpoint in trial 2, hypertension: “The proportion of patients with satisfactorily controlled blood pressure according to recommended targets: under 140/90 mmHg in patients aged under 80 years with hypertension; […]” | Potentially related to one of the components of the primary endpoint in trial 1, diabetes: “The proportion of patients with type 2 diabetes achieving […] BP below 140/80 mmHg”. |
| **Stroke and transient ischemic attack** | **STIA003** | The percentage of patients with a history of stroke or TIA in whom the last blood pressure reading (measured in the preceding 12 months) is 150/90 mmHg or less. | Related to one of the components of the primary endpoint in trial 2, hypertension: “The proportion of patients with satisfactorily controlled blood pressure according to recommended targets: […] under 140/90 mmHg in patients aged under 80 years with a history of stroke/transient ischemic attack […]” | Potentially related to one of the components of the primary endpoint in trial 1, diabetes: “The proportion of patients with type 2 diabetes achieving […] BP below 140/80 mmHg”. |
| **Diabetes mellitus** | **DM002** | The percentage of patients **with diabetes**, on the register, in whom the last blood pressure reading (measured in the preceding 12 months) is 150/90 mmHg or less. | Related to one of the components of the primary endpoint in trial 1, diabetes: “The proportion of patients with **type** 2 diabetes achieving all three of the following treatment targets: blood pressure below 140/80 mmHg (or 130/80 mmHg if there is kidney, eye or cerebrovascular damage), […]” and in trial 2, hypertension: “The proportion of patients with satisfactorily controlled blood pressure […] BP < 140/80 in patients aged under 80 years with diabetes, and <130/80 if there are complications of diabetes, […]. |  |
| **Diabetes mellitus** | **DM003** | The percentage of patients **with diabete**s, on the register, in whom the last blood pressure reading (measured in the preceding 12 months) is 140/80 mmHg or less. | Related to one of the components of the primary endpoint in trial 1, diabetes: “The proportion of patients with **type** 2 diabetes achieving all three of the following treatment targets: blood pressure below 140/80 mmHg (or 130/80 mmHg if there is kidney, eye or cerebrovascular damage), […]” and in trial 2, hypertension: “The proportion of patients with satisfactorily controlled blood pressure […] BP < 140/80 in patients aged under 80 years with diabetes, and <130/80 if there are complications of diabetes, […]. |  |
| **Diabetes mellitus** | **DM004** | The percentage of patients with diabetes, on the register, whose last measured total serum cholesterol (measured within the preceding 12 months) is 5 mmol/l or less. | Related to one of the components of the primary endpoint in trial 1, diabetes: “The proportion of patients with type 2 diabetes achieving all three of the following treatment targets: blood pressure below 140/80 mmHg (or 130/80 mmHg if there is kidney, eye or cerebrovascular damage), HbA1c value below or equal to 59 mmol/mol and total serum cholesterol level below or equal to 5.0 mmol/l” |  |
| **Diabetes mellitus** | **DM006** | The percentage of patients with diabetes, on the register, with a diagnosis of nephropathy (clinical proteinuria) or micro-albuminuria who are currently treated with ACE-I (or ARBs). | Related to one of the components of the primary endpoint in trial 1, diabetes: “The proportion of patients with type 2 diabetes achieving all three of the following treatment targets: blood pressure below 140/80 mmHg (or 130/80 mmHg if there is kidney, eye or cerebrovascular damage), HbA1c value below or equal to 59 mmol/mol and total serum cholesterol level below or equal to 5.0 mmol/l” |  |
| **Diabetes mellitus** | **DM007** | The percentage of patients with diabetes, on the register, in whom the last IFCC-HbA1c is 59 mmol/mol or less in the preceding 12 months. | Related to one of the components of the primary endpoint in trial 1, diabetes: “The proportion of patients with type 2 diabetes achieving all three of the following treatment targets: blood pressure below 140/80 mmHg (or 130/80 mmHg if there is kidney, eye or cerebrovascular damage), HbA1c value below or equal to 59 mmol/mol and total serum cholesterol level below or equal to 5.0 mmol/l” |  |
| **Diabetes mellitus** | **DM008** | The percentage of patients with diabetes, on the register, in whom the last IFCC-HbA1c is 64 mmol/mol or less in the preceding 12 months. | Related to one of the components of the primary endpoint in trial 1, diabetes: “The proportion of patients with type 2 diabetes achieving all three of the following treatment targets: blood pressure below 140/80 mmHg (or 130/80 mmHg if there is kidney, eye or cerebrovascular damage), HbA1c value below or equal to 59 mmol/mol and total serum cholesterol level below or equal to 5.0 mmol/l” |  |
| **Diabetes mellitus** | **DM009** | The percentage of patients with diabetes, on the register, in whom the last IFCC-HbA1c is 75 mmol/mol or less in the preceding 12 months. | Related to one of the components of the primary endpoint in trial 1, diabetes: “The proportion of patients with type 2 diabetes achieving all three of the following treatment targets: blood pressure below 140/80 mmHg (or 130/80 mmHg if there is kidney, eye or cerebrovascular damage), HbA1c value below or equal to 59 mmol/mol and total serum cholesterol level below or equal to 5.0 mmol/l” |  |
| **Diabetes mellitus** | **DM0012** | The percentage of patients with diabetes, on the register, with a record of a foot examination and risk classification: (1) low risk (normal sensation, palpable pulses), (2) increased risk (neuropathy or absent pulses), (3) high risk (neuropathy or absent pulses plus deformity or skin changes in previous ulcer) or (4) ulcerated foot within the preceding 12 months. | Related to one of the secondary endpoints in trial 1, diabetes: “The effects on recorded processes of care (the proportion of patients with type 2 diabetes achieving all nine of the following recommended processes of care in the previous 12 months: blood pressure recording, HbA1c recording, total serum cholesterol recording, urine albumin to creatinine ratio (ACR) or protein to creatinine ratio (PCR) or proteinuria coding, estimated glomerular filtration rate (eGFR) or serum creatinine testing, foot care review, retinal screening, body mass index recording, smoking status)” |  |
| **Diabetes mellitus** | **DM0014** | The percentage of patients newly diagnosed with diabetes, on the register, in preceding 1 April to 31 March who have a record of being referred to a structured education programme within 9 months after entry on to the diabetes register | Related to the denominator of the primary and secondary endpoints in trial 1, diabetes. |  |
| **Diabetes mellitus** | **DM0018** | The percentage of patients with diabetes, on the register, who have had influenza immunisation in the preceding 1 August to 31 March. | Related to the denominator of the primary and secondary endpoints in trial 1, diabetes. |  |

**Table 4: Non trial-related QOF Indicators 2015-16**

| **Domain** | **QOF indicator number** (2015–16) | **Indicator** |
| --- | --- | --- |
| **Secondary prevention of coronary heart disease** | **CHD005** | The percentage of patients with coronary heart disease with a record in the preceding 12 months that aspirin, an alternative anti-platelet therapy or an anti-coagulant is being taken. |
| **Secondary prevention of coronary heart disease** | **CHD007** | The percentage of patients with coronary heart disease who have had influenza immunisation in the preceding 1 August to 31 March. |
| **Mental health** | **MH002** | The percentage of patients with schizophrenia, bipolar affective disorder and other psychoses who have a comprehensive care plan documented in the record, in the preceding 12 months, agreed between individuals, their family and/or carers as appropriate. |
| **Mental health** | **MH003** | The percentage of patients with schizophrenia, bipolar affective disorder and other psychoses who have a record of blood pressure in the preceding 12 months. |
| **Smoking** | **SMOK002** | The percentage of patients with any or any combination of the following conditions: coronary heart disease (CHD), peripheral arterial disease (PAD), stroke or transient ischemic attack (TIA), hypertension, diabetes, chronic obstructive pulmonary disease (COPD), chronic kidney disease (CKD), asthma, schizophrenia, bipolar affective disorder or other psychoses whose notes record smoking status in the preceding 12 months. |
| **Smoking** | **SMOK004** | The percentage of patients aged 15 or over who are recorded as current smokers who have a record of an offer or support and treatment within the preceding 24 months. |
| **Smoking** | **SMOK005** | The percentage of patients with any or any combination of the following conditions: CHD, PAD, stroke or TIA, hypertension, diabetes, COPD, CKD, asthma, schizophrenia, bipolar affective disorder or other psychoses who are recorded as current smokers who have a record of an offer of support and treatment within the preceding 12 months |
| **Asthma** | **AST003** | The percentage of patients with asthma, on the register, who have had an asthma review in the preceding 12 months that includes an assessment of asthma control using the three Royal College of Physician questions. |

### 2.2.2 Other analyses

- To assess fidelity of intervention delivery as measured by the number of audit and feedback reports received, completion of fidelity survey, take-up of practice outreach visits, development of action plans and acceptance / take-up of computerised prompts / searches.
- To explore the relationship between intervention fidelity and intervention effects, summarising the change in adherence between baseline and follow-up for the primary endpoints for each measured aspect of fidelity listed above.
- To assess whether intervention effects vary over time for the primary endpoint using practice achievement at baseline and from the four audit and feedback reports.
- To explore the Hawthorne effect replacing practice achievement data in the control arms with that from the randomised non-intervention group in a repeat of the primary endpoint analysis.
- To explore the relationship between the primary endpoints and the Care Quality Commission (CQC) ratings as measured by the proportion of patients adhering to allocated recommendations at baseline and at follow-up by overall CQC rating and the 5 key questions asked in CQC inspections.
- To explore the differences between trial and process evaluation practices, by comparing baseline characteristics and achievement of primary and secondary outcomes.

## 2.3 Derivation of endpoints and other data

All individual indicators (secondary endpoints) that contribute to the composite indicators (primary endpoints) as well as comorbidity and polypharmacy are derived by CSU in Wakefield using medical records in SystmOne. Detailed documentation of the database operations used to derive the endpoints, including clinical read codes, can be found in P:\CTRU\Projects\Health_Sciences\Complex Interventions\ASPIRE\TC\14_DataManagement\14.5 CSU Searches\Final. Composite indicators, list size (categorical variable) and pre-intervention achievement are derived by the trial statistician at CTRU. SAS programs that derive composite outcomes (primary endpoints) as described in Table 2 will be reviewed by the supervising statistician.

During randomisation, list size, pre-intervention achievement and overall QOF achievement were defined as above / below specified cut-points (see below) and summaries of stratification factors will use these cut-points, however in the main analysis the actual baseline values will be used as covariates:

- List size is defined as small (below the median) and large (above the median) using the median list size of West Yorkshire practices (6562).
- Pre-intervention achievement in relevant recommendations is categorised into low (below the median) and high (above the median) using the medians of relevant pre-intervention achievement of recruited practices in the respective trial.
- Overall QOF achievement is defined as a categorical variable (below and above median) based on the overall achievement score in the trial period (QOF year 2014-2015).

Patient ages are categorised as follows: <55, 55-64, 65-74, 75-84 and 85+.

Comorbidity will be calculated for each participant by summing the patient's appearance on 20 appropriate QOF registers from the 2015/16 QOF year (Arterial Fibrillation, Asthma, Cancer, Chronic Kidney Disease, Chronic Obstructive Pulmonary Disease, Coronary Heart Disease, Cardiovascular Disease, Dementia, Depression, Diabetes, Epilepsy, Heart Failure, Hypertension, Learning Disabilities, Mental Health, Obesity, Osteoporosis, PAD, Stroke and Thyroid).

Polypharmacy is defined as the number of current repeat prescriptions on a patient’s electronic patient health record.

The number of GP partners will be derived from data obtained from NHS Digital and will be calculated as follows:

Total number of GP Partners = Total number of GPs – Total number of salaried GPs

## 2.4 Missing data

The completeness of the data depends on the completeness of the medical records in SystmOne and cannot be assessed within the study dataset. Missing read codes can lead to patients incorrectly being included in or excluded from the denominator of a specific clinical recommendation and also to a patient incorrectly achieving or not achieving a recommendation. For the primary and secondary analyses we assume that if data is missing it is missing at random or at least that missingness of data is not related to the study interventions or outcomes. If these assumptions hold true, estimates of the primary endpoints will be unbiased.

The extent of missing data (at both the participant and practice level) at baseline and outcome will be assessed and reasons for missingness investigated. Due to the method of randomisation / stratification there will be no missing baseline data at the GP practice level for list size, CCG and pre-intervention achievement. It is possible however that QOF achievement at the practice level or sex, age, number of co-morbidities and polypharmacy at the patient level may not be available in all cases. In the case where QOF achievement is missing, we will use other available data from the practice, in combination with data from other practices to impute the missing score e.g. practice list size, teaching status of practice etc. If patient level (sex, age, number of comorbidities or polypharmacy) data are missing the mean of the observed values within the practice will be imputed.

Our interventions may incentivise general practices to remove patients from the denominator of certain recommendations in order to improve achievement. In this case above assumptions regarding missingness do not hold true and the endpoint estimates will be upward biased, showing an intervention effect that is larger than the true effect. We will use data from the previous data extractions (randomisation/stratification and audit & feedback reports) to calculate trends in the denominator for each primary endpoint (aggregated by arm and standardised, with the randomisation/stratification data being the base of the index (100%)). We will plot these trends by arm for each primary endpoint. These trends are expected to be fairly similar in both arms. If we observe any substantial differences, we will include the relative change of the denominator between baseline and follow-up ((Denominator_follow-up_-Denominator_fbaseline_)/Denominator_baseline_) as a practice-level covariate in any analysis.

General practices for which all (follow-up) data are missing (i.e. have baseline but no further data) are excluded from the primary analysis. All data might be missing for instance if a practice merged with another practice that is not participating in ASPIRE or if a practice ceased to exist prior to the download for the first audit and feedback report. The number of practices which are excluded from the primary analysis due to this reason will be summarised overall and by arm. If practices are excluded from the primary analysis because of this reason, we will perform a sensitivity analysis using multiple imputation to predict the missing outcome data based upon the available data of other practices.

General practices for whom final outcome data is missing but audit and feedback data is available will be included in the primary analysis. Data may be missing if a practice merged with another non-ASPIRE practice or closed following the download for the first audit and feedback report. The number of practices with incomplete final outcome data will be summarised. If any practice fulfils this criteria, we will use available data from prior audit and feedback reports to impute the final outcome data for the practice. A sensitivity analysis using practice characteristics and multiple imputation may be conducted if a large number of practices fit this criteria.

If a participating practice (A) merges with another participating practice (B) (practice A ceases to exist and practice B encompasses all patients from practice A), we will still be able to obtain the vast majority of relevant patient records that is needed to assess the primary outcome for both original practices and we can link these records to the original practices using data from the baseline assessment or the audit and feedback reports. Patients originally registered with practice A will be assigned to practice A, all other patients (both those originally registered to practice B and those newly registered / born since the merge) will be assigned to practice B.

CSU provides the last recorded measurement in SystmOne for each patient. However, if a patient had two or more measurements recorded on that day, all measurements are received in the outcome dataset. If this occurs for any patient, the mean of the non-missing values of all recorded measurements on that day will be used in the analysis.

If implausible values for continuous intermediate clinical outcomes are observed, these will be set to missing in the analysis. The following intervals are defined as plausible values:

- Diastolic blood pressure: 20 mmHg - 150 mmHg
- Systolic blood pressure: 50 mmHg - 250 mmHg
- HbA1c: 10 mmol/mol - 170 mmol/mol
- Total Serum Cholesterol: 0.5-12 mmol/l.

## 2.5 Non-standard circumstances

The co-primary endpoints (achievement of recommendations) will be assessed at a fixed point in time (31st March 2016). However, achievement rates on this day reflect the GPs’ behaviour over a period of up to 11 months. In the analysis, patient observations will be linked to the practice that the patients are registered with, at the time of the final download (according to the practice code in the patients’ SystmOne electronic health record) even if they have changed their GP during the course of the trial. The exception are patients who are, at follow-up, registered with a practice that has merged with another participating practice. These patients will be analysed as if they were registered with the original practices (see section 2.4).

Baseline data (sex and age) from patients who moved from a participating to a non-participating practice (including practices outside of the study area) or who died during the course of the trial will still be used as covariates in the final analysis for the practice to which they were originally registered.

# 3. Populations

## 3.1 Eligibility

General practices are eligible if they use the *SystmOne* computerised clinical system (TPP, <http://www.tpp-uk.com/>). Approximately two thirds of West Yorkshire practices use *SystmOne*. The use of a single system simplifies the process of data extraction and of implementing software support. Practices are excluded if they were involved in earlier stages of intervention development and piloting. Practices in one CCG involved in a concurrent initiative addressing anticoagulation in atrial fibrillation are excluded from Trial 2.

## 3.2 Intention to treat population

All analysis and data summaries will be conducted on the Intention to Treat Population (ITT). All relevant patients in randomised GP practices for which data are available will be included and analysed as if they received the intervention that they were allocated to, irrespective of what intervention their GP practice has actually received and irrespective of whether their GP practice has received any intervention at all.

## 3.3 Screening population

The screening population will consist of all practices screened for eligibility.

## 3.4 Sensitivity analysis population

In case that all follow-up data is missing for one or several GP practices, we will perform a sensitivity analysis using multiple imputation as described in section 2.4. The number, age and sex of patients for whom missing values have to be imputed will be taken from baseline or audit and feedback datasets (latest available data).

# 4. Data Handling

## 4.1 Data monitoring

For a cluster trial of this nature and duration, a separate Data Monitoring and Ethics Committee is not required. Rather, the TSC will adopt a safety monitoring role, with the constitution of a sub-committee to review safety issues where this becomes necessary.

## 4.2 Data validation

### 4.2.1 CSU Data

All derivations of the secondary endpoints are calculated at CSU in Wakefield. Chris Jackson (CSU Wakefield), Thomas Willis (LIHS) and John Turgoose (CTRU) have validated the derivations of the primary endpoints by reviewing the documented search pdfs.

Prior to analysis, SAS will be used to validate the data and identify any inconsistent or missing data. Checks to be performed include:

1. data is available for all randomised practices
2. data is available for all primary and secondary endpoints
3. dataset is coherent (indicators that should have the same number of patients in the denominator (e.g. all Diabetes Type II patients) actually have the same number of patients in denominator).

Any issues identified by the data cleaning programme will be investigated and resolved with CSU prior to any analysis being conducted.

### 4.2.2 Outreach Visit Data

CRFs will be returned to the CTRU for data entry. Data will be downloaded and read into permanent SAS data sets. The names and contents of the variables can be found in the annotated final CRF specification in P:\CTRU\Database Documentation\ASPIRE.

SAS will be used to validate the data and identify any inconsistent or missing data. Checks to be performed include:

- Checks for unusual and outlying data
- Checks for inconsistent data
- Checks for missing data
- Other checks as deemed appropriate

Any suspicious or inconsistent data identified via these checks will be noted and the Data Manager will be notified by email. The Data Manager or their delegate will check such inconsistencies against the CRFs and if there has been an error in data input causing such inconsistencies this will be corrected on the database.

### 4.2.3 Other data

Data related to screening, Audit and Feedback report distribution, QOF (individual indicators), organisational groups, fidelity survey and CQC ratings will be imported directly into SAS. SAS will be used to validate the data as per section 4.2.2 however as this data is not present on paper CRFs and is downloaded from the internet, it will not be possible to query data therefore any inconsistencies will be noted but are unlikely to be resolved.

# 5. Data Analysis

Statistical analysis is the responsibility of the CTRU Trial Statistician. The final analysis will be conducted on the ITT population unless otherwise stated.

## 5.1 General calculations

All analyses will be carried out using SAS version 9.4 unless stated otherwise. All percentages will include subjects with missing data in the denominator wherever possible however, due to technical reasons, this information is unlikely to be available for many of the primary and secondary outcomes (see section 2.4).

Percentages, means, medians and ranges will be reported with two decimal places. If not stated otherwise, all statistical tests will be two-sided using an alpha of 0.025. P-values will be reported to three decimal places.

## 5.2 Study summary

A study summary will contain details of the ASPIRE milestones, screening, recruitment, randomisation, withdrawals and follow up; from which an overall CONSORT diagram appropriate for cluster randomised trials will be produced.

### 5.2.1 Milestones

The ASPIRE recruitment period, follow up period, and data cut-off points for the final report will be summarised.

### 5.2.2 Screening

These summaries will use the screened population as defined in section 3.3. Practice eligibility, recruitment and randomisation will be summarised overall and by CCG and will include: the number of potentially eligible practices, the number eligible for trial entry and the number not opting out. Reasons for non-entry into ASPIRE will also be presented overall and will include: reasons ineligible and reason for opt-out. In addition, the time to opt-out for all eligible practices will be summarised.

Demographics of all screened practices will be summarised and will include number of registered patients, number of salaried GPs per practice, number of GP partners per practice, total number of GPs, deprivation score (Index of Multiple Deprivation), % of patients who would recommend the practice, total QOF points (2014/2015), % of patients who saw / spoke to a nurse / GP on the same or next day, whether or not a practice is a teaching practice and CCG. The characteristics of all screened practices will be compared to those randomised.

### 5.2.3 Practice withdrawals / mergers / closures

The number and timing of practice withdrawals, mergers and closures will be summarised by trial and recommendation and where possible the reasons for withdrawal will be presented.

### 5.2.4 Baseline characteristics

Stratification factors (list size, CCG and pre-intervention adherence) and baseline patient characteristics (age and gender) will be summarised using frequencies and summary statistics by trial and recommendation. Missing or unobtainable data will be included as missing and no statistical testing will be carried out on these data.

Baseline patient and practice characteristics will also be summarised for control and process evaluation practices.

## 5.3 Primary endpoint analysis

As the trials are cluster randomised, the primary outcome measures, adherence to the four recommendations, will each be compared between the intervention and control groups using two-level binary logistic models, with patients nested within general practices for each composite outcome at 11 months post randomisation. Analyses will be adjusted for the GP-level stratification factors used for randomisation (list size, CCG and pre-intervention achievement in the relevant recommendations), the overall baseline (2014/15) QOF achievement score at the practice level (single value) and sex, age, number of comorbidities and polypharmacy (number of current repeat prescriptions) at the patient level. Regression diagnostics will be used to check that the models do not violate their underlying assumptions. Effect sizes and 97.5% confidence intervals will be reported.

## 5.4 Secondary endpoint analysis

##

### 5.4.1 Intervention effects on separate indicators that make up the composite outcomes

Analysis for the separate indicators that make up the composite outcomes will exactly follow the primary endpoint analysis as described in section 5.3.

### 5.4.2 Intervention effects on recorded processes of care

Analysis for the recorded processes of care will exactly follow the primary endpoint analysis as described in section 5.3.

### 5.4.3 Intervention effects on continuous intermediate clinical outcomes

Two-level linear models, with patients nested within general practices will be used to compare the outcomes at 11 months post randomisation. Analyses will be adjusted for stratification factors used for randomisation (list size, CCG and pre-intervention achievement in the relevant recommendations), the overall baseline (2014/15) QOF achievement score at the practice level (single value) and sex, age, number of comorbidities and polypharmacy (number of current repeat prescriptions) at the patient level. Regression diagnostics will be used to check that the models do not violate their underlying assumptions. Effect sizes and 97.5% confidence intervals will be reported.

### 5.4.4 Intervention effects on relevant QOF indicators

As patient level data is unavailable, the proportion of patients adhering to any given QOF indicator (see Tables 3 and 4) for each GP practice will be used in a linear model to explore the effect of the intervention on relevant QOF indicators. Data will be extracted for the 2015-2016 QOF year to allow us to assess outcomes at 11 months post randomisation. This analysis is explorative and is not powered to detect any differences. Effect sizes and p-values will be reported but will be interpreted with caution.

### 5.4.5 Fidelity of intervention delivery

Qualitative summaries of intervention delivery are not covered in this analysis plan but will be included in the final NIHR report. Quantitative data summaries of intervention delivery are detailed below.

#### Audit and Feedback reports

The number of Audit and Feedback reports sent to practices will be summarised overall, by trial arm by time-point and by method of distribution (post / email) at each time-point.

Implementation fidelity will be further assessed via results from a brief questionnaire emailed to the practices after the last A&F reports are circulated. The survey will include the following questions:

1. Did you use the ASPIRE SystmOne searches on (#clinical topic) (Yes / No)
2. Do you remember receiving ASPIRE audit reports on (#clinical topic)? (Yes/No)
3. If yes, were the audit reports:
   1. Relevant to the practice? (Yes/No)
   2. Shared with colleagues in the practice? (Yes/No)
   3. Discussed within the practice? (Yes/No)
   4. Used to change how people in the practice worked? (Yes/No)
4. What is your role?
5. Do you have any further comments on the ASPIRE interventions?

Practices allocated to the Risky Prescribing intervention were also asked the following question:

1a. Did you use the ASPIRE SystmOne protocols (prompts)?

The responses to Q1 will be recoded as: Yes=Received, No=Not received. The responses to Q3a and Q3b will be recoded as: Yes=medium engagement, No=little or no engagement and responses to Q3c and Q3d will be recoded as: Yes=High engagement, No=little or no engagement.

The number of surveys emailed and completed will be summarised. Item values will be summarised by trial and recommendation.

#### Outreach visits

Practice outreach visits are planned to occur between 3-6 months after delivery of the baseline audit and feedback report and are scheduled to last approximately 30 minutes. During the visit, the facilitator discusses the report and assists the attendees in production of a relevant action plan. It is therefore intended that the clinical lead attend this visit as a minimum. Practices who take up the first outreach visit are also offered the opportunity to have a second follow-up visit later in the trial.

Fidelity of delivery of the outreach visit will be assessed by summarising whether or not an outreach visit took place, when the visit took place and who delivered it, the length of the outreach visit in minutes, the number of members of practice staff attending the visit, including whether or not key leaders were present, and whether content was delivered as planned by trial and recommendation. The number of practices taking up the offer of a second outreach visit will also be summarised. Where available, the reasons for non-take up of outreach visits will also be presented. Practice performance for the primary outcomes will also be summarised by whether or not an outreach visit took place. The number of practices participating in other quality improvement initiatives, together with details of these initiatives will also be summarised by trial and recommendation.

#### Action plans

Fidelity of delivery of the action plans will be assessed by summarising whether or not an action plan was developed (either before or during the outreach visit) and received by the research team, by trial and recommendation.

#### Computerised prompts / searches

Fidelity of delivery of the searches will summarise the number of practices who joined the organisational groups from which they could download the searches into their system. For those practices randomised to the Risky Prescribing recommendation, fidelity of delivery of the computerised prompts will be presented in the same way. It is important to note here that membership of the organisational group does not imply usage of the prompts / searches and there are known technical difficulties with group acceptance data.

#### Relationship between intervention fidelity and intervention effects

To explore the relationship between intervention fidelity and the primary outcomes we will present the mean change in the proportion of patients adhering to the allocated recommendation at baseline and at follow-up, by recommendation for the following:

- Number of audit and feedback reports received (1,2,3,4)
- Whether or not a practice responded to the email survey (y/n)
- Whether or not an action plan was developed (y/n)
- Whether or not a practice joined the appropriate organisational group (y/n)

To further investigate whether any of the intervention fidelity measures described above influence outcomes, formal mediation analysis may be appropriate. Mediator analysis explores the extent to which the intervention effect can be explained by an intermediate mechanistic outcome which in turn can help us to judge which aspects of the intervention may be further enhanced to optimise benefit. The proposed mediators are post randomisation effect modifiers i.e. process variables which are aspects involved in the actual delivery of the intervention. Formal mediation analysis is outside of the scope of this analysis plan however if data quality is sufficient (to be determined as a result of summaries detailed in section 5.4.5), and suitable techniques appropriate for cluster randomised trials are identified then a separate analysis plan detailing such formal mediation analysis will be produced.


### Time-varying effects

Data extracted pre-intervention (for randomisation/stratification) and during the trial (as part of the 4 audit and feedback intervention reports) for the primary endpoints listed in Table 2 will be included in an exploratory repeated measures analysis, to estimate differences in intervention effects between the treatment groups over time.

This will involve mixed model analysis, which allows for randomised arm, time effects, baseline achievement, and treatment-time interactions (fixed effects). A covariance pattern model will be used which takes account of the fact that repeated measures on the same GP practice may be correlated, by modelling the pattern of covariance between each of the repeated observations. To allow for the way in which successive assessments of achievement are correlated first-order heterogeneous autoregressive covariance matrix will be used. This covariance matrix requires fewer parameters than the unstructured covariance matrix, whilst allowing variability to differ between assessments of achievement, and covariance’s to decrease exponentially depending on their distance from baseline (i.e. allowing for the expectation that achievement at baseline will have a greater association with achievement at the time of the first audit and feedback report than achievement at baseline with the final audit and feedback report).

Model assumptions will be checked using Pearson and Studentised residuals. The mean differences between the arms within each trial and 95% Confidence intervals (CIs) will also be presented for each time-point, as will boxplots showing means and 95% CIs, and medians and ranges.

### Exploring the Hawthorne effect

We will use the group of practices that have not opted out from participation in the trial but were randomised to act as a non-intervention control to test for the existence of a Hawthorne effect. We will repeat all primary analyses replacing the original control arm with the non-intervention controls. If there is a Hawthorne effect, we expect the intervention effects to be larger in this repeat analysis. According to the theory, if a Hawthorne effect is noted, the non-random part of the differences in the intervention effects can be attributed to the fact that practices are aware of being observed (and this is why they improve adherence to recommendations) and is not attributable to the intervention. The Hawthorne effect is the key reason for the use of the incomplete block design in ASPIRE.

### Relationship between Care Quality Commission ratings and primary outcomes

The Care Quality Commission monitors, inspects and regulates practices to ensure they meet fundamental standards of quality and safety. Five key questions are asked during inspections and these are shown in Table 5 below. Practices are rated as outstanding, good, requires improvement or inadequate. We will explore the relationship between these ratings and the primary outcomes, and the overall practice rating.

The number of practices with a CQC rating at either baseline or follow-up or at both time-points will be reported. The timing of the CQC inspection will be plotted for all practices in relation to baseline and delivery of the 4 audit and feedback reports. Practice ratings will be summarised overall and by key question for all practices overall, by trial and recommendation.

To explore the relationship between CQC ratings and the primary outcomes we will present the mean change in the proportion of patients adhering to the allocated recommendation at baseline and at follow-up, by overall CQC rating and the five key questions of interest.

**Table 5: Key questions asked in CQC inspections**

| Five key questions | How defined |
| --- | --- |
| Q1 - Are they safe? | Protected from abuse and avoidable harm. |
| Q2 - Are they effective? | People’s care, treatment and support achieves good outcomes, promotes a good quality of life and is based on the best available evidence. |
| Q3 - Are they caring? | Staff involve and treat people with compassion, kindness, dignity and respect. |
| Q4 - Are they responsive to people’s needs? | Services are organised so that they meet people’s needs. |
| Q5 - Are they well-led? | Leadership, management and governance ensures delivery of high-quality person-centred care, supports learning and innovation, and promotes an open and fair culture. |

### To explore the relationship between achievement and engagement for process evaluation practices

The baseline characteristics and outcomes of the process evaluation sites will be compared to trial practices, overall, by trial and recommendation to investigate whether there are any systemic differences between trial and process evaluation practices. In addition, we will examine the relationship between achievement and qualitative engagement data for process evaluation practices.

## 5.5 Sensitivity analysis

### 5.5.1 Missing data at patient level

The number of patients with missing data at baseline and follow-up will be presented. If patient level data is missing, we will use data from previous data transfers to test our hypothesis that it is missing at random, as outlined in section 2.4.

### 5.5.2 Missing data at practice level

The number of practices where all follow-up data is missing will be presented. In the case that all follow-up data is missing for one or several GP practices, we will perform a sensitivity analysis of the primary analysis using imputation as described in section 2.4. The number, age and sex of patients for whom missing values have to be imputed will be taken from baseline or audit and feedback datasets (latest available data).

# 6. Reporting and Dissemination of the Results

A full statistical report of the analysis following the template laid out in this final analysis plan will take place, and where possible the statistical report will take the format appropriate for the NIHR Journals Library. It is estimated that this will take approximately six months from the final import of CSU data. After these analyses are complete, the results will be presented to the project teams. The members of the project team will then write up the results into manuscript(s) for submission to a peer-reviewed journal(s) as per the ASPIRE publication plan and will submit a monograph/end of programme report to the funder as per the terms of the trial contract.

The results will also be submitted as abstracts to appropriate conferences for either poster or oral presentation. Details outlining the principles to be followed for the preparation, review, authorship and publication of abstracts and manuscripts can be found in the ASPIRE Publication Policy section of the protocol Version 3.0 (02/02/2015).

# 7. References

1. Craig P, Dieppe P, Macintyre S, Michie S, Nazareth I, Petticrew M: **Developing and evaluating complex interventions: the new Medical Research Council guidance.** *BMJ* 2008, 337:a1655.
2. Grimshaw JM, Thomas RE, MacLennan G, Fraser C, Ramsay CR, Vale L, Whitty P, Eccles MP, Matowe L, Shirran L, et al: **Effectiveness and efficiency of guideline dissemination and implementation strategies.** *Health Technol Assess* 2004, 8.
3. Eccles M, Grimshaw JM, Campbell M, Ramsay C: **Research designs for studies evaluating the effectiveness of change and quality improvement strategies.** *Qual Saf Health Care* 2003, 12:47-52.
4. Grimshaw JM, Eccles MP, Lavis JN, Hill SJ, Squires JE: **Knowledge translation of research findings.** Implementation Science 2012, **7**:50
5. Jamtvedt G, Young JM, Kristoffersen DT, O'Brien MA, Oxman AD: **Audit and feedback: effects on professional practice and health care outcomes.** *Cochrane Database of Systematic Reviews* 2006
6. 48 Eccles M, Steen N, Grimshaw J, Thomas L, McNamee P, Soutter J, Wilsdon J, Matowe L, Needham G, Gilbert F, Bond S: **Effect of audit and feedback, and reminder messages on primary-care radiology referrals: a randomised trial.** Lancet 2001, 357:1406-1409.
7. Thomas RE, Croal BL, Ramsay C, Eccles M, Grimshaw J: **Effect of enhanced feedback and brief educational reminder messages on laboratory test requesting in primary care: a cluster randomised trial.** Lancet 2006, 367:1990-1996.
8. Eccles MP, McColl E, Steen N, Rousseau N, Grimshaw J, Parkin D, et al: **Effect of computerised evidence based guidelines on management of asthma and angina in adults in primary care: cluster randomised controlled trial.** BMJ 2002, 325:941-944.
9. Eccles M, Whitty PM, Speed C, ISteen IN, Vanoli A, Hawthorne GC, Grimshaw JM, Wood LJ, McDowell D: **A pragmatic cluster randomised controlled trial of a Diabetes REcall And Management system: the DREAM trial.** Implementation Science 2007, 2:6.
10. Rogers EM: **Diffusion of Innovations**. New York: Free Press; 1995.
11. White IR, Carpenter J, Horton NJ: **Including all individuals is not enough: lessons for intention-to-treat analysis**. Clin Trials 2012, 9:396-407
12. Pituch KA, Stapleton LM, Kang JY: **A comparison of single sample and bootstrap methods to assess mediation in cluster randomized trials**. Multivariate Behavioural Research 2010:367-400.
