# Supplementary material for: An adaptable implementation package targeting evidence-based indicators in primary care: A pragmatic cluster-randomised evaluation
Source: PLoS Med. 2020 Feb 28;17(2):e1003045. doi: 10.1371/journal.pmed.1003045 (PMC7048270; doi:10.1371/journal.pmed.1003045)
Supplement: Attachment — Submitted filename: PMEDICINE-D-19-02682 response to reviewers 191128.docx [file pmed.1003045.s014.docx]

Response to reviews

**An adaptable implementation package targeting evidence-based indicators in primary care: a pragmatic cluster-randomised evaluation**

**PMEDICINE-D-19-02682**

We thank the editors and reviewers for helpful feedback on this manuscript. We believe that we have now been able to improve the manuscript in light of this feedback.

**Requests from the editors**

1. Competing Interest Statement: Please clarify the role that DP, PC and SJ had in the implementation and analyses of the study, in light of their associations with NHS Bradford Districts CCG, and Prescribing Support Services, Ltd.

Further clarification has been added to the ‘Competing Interests’ section at the end of the manuscript:

*DP was responsible for the recruitment and training of pharmacist-facilitators to deliver the educational outreach visits. Neither he nor the facilitators were involved in the analysis. PC and SJ were not involved in the implementation or analysis of the study.*

2. Data Availability Statement: PLOS Medicine requires that the de-identified data underlying the specific results in a published article be made available, without restrictions on access, in a public repository or as Supporting Information at the time of article publication, provided it is legal and ethical to do so. Please see the policy at

<http://journals.plos.org/plosmedicine/s/data-availability> and FAQs at <http://journals.plos.org/plosmedicine/s/data-availability#loc-faqs-for-data-policy>

Please include an appropriate contact (web and/or email address) for inquiries to obtain access to the data (please note: this cannot be a study author).

We have replaced the original contact provided here (a study author) with the data access contact at the Leeds Institute of Clinical Trials Research.

3. Abstract Background: The final sentence should clearly state the study question/hypothesis to be tested.

We have clarified the study question.

*We compared an adaptable implementation package against usual practice and assessed effects on adherence to four different evidence-based indicators.*

4. Abstract: Methods and Findings: Please define the control for the intervention.

We have clarified the nature of each comparison with the incomplete block trial design.

*Within trials, each arm acted as the usual practice comparison for the other targeted indicator. For example, practices assigned to the diabetes control package acted as the usual practice comparison for practices assigned to the risky prescribing package.*

5. Abstract: Methods and Findings (Line 120): You mention here that 34 practices were recruited as non-intervention controls. This could be misleading because you do not include any analyses of these controls in this manuscript, and comparisons are made to within-arm controls. Please remove the mention of these non-intervention controls here, and clarify the control for these analyses

We had originally included the 34 non-intervention practices in the interests of transparency. We agree that their inclusion in the abstract may confuse readers, especially if they are not included in the trial analyses we present. We have clarified the controls for the trial comparisons above.

6. Abstract: Methods and Findings: In the last sentence of the Abstract Methods and Findings section, please describe the main limitation(s) of the study's methodology

We have added the main limitations of the study methods, focusing on two we consider as the most important.

*Two key study limitations concern our inability to make any inferences about the relative effects of individual intervention components, given the multifaceted nature of the implementation package, and that the composite endpoint for diabetes control may have been too challenging to achieve.*

7. Abstract: Conclusions: Please interpret the study based on the results presented in the abstract, emphasizing what is new without overstating your conclusions; the phrase "In this study, we observed ..." may be useful. Please avoid vague statements such as "these results have major implications for policy/clinical care". Mention only specific implications substantiated by the results. Specifically, both sentences of the conclusion are vague and do not clearly reflect what can be concluded from the study findings.

We have specified our conclusion as follows:

*In this study, we observed that a multifaceted implementation package was relatively cost-effective for targeting prescribing behaviours within the control of clinicians but not for more complex behaviours that also required patient engagement.*

8. Author Summary: Thank you for including an Author Summary. The Author Summary should immediately follow the Abstract in your revised manuscript.

We have moved the Author Summary as requested.

9. Author Summary: “What do these findings mean?”: Please revise the final point to: “In this study, we found that a ‘one-size-fits-all’ strategy did not work…” Please also revise this point to clarify what is meant by “did not work” and “the nature of clinical behaviors targeted”.

We have now changed this.

*In this study, we found that an adaptable implementation package was relatively cost-effective for targeting prescribing behaviours within the control of clinicians but not for more complex behaviours that also required patient engagement.*

10.Introduction: Please conclude the Introduction with a clear description of the study question or hypothesis.

We have now specified the study objective:

*We compared the effects of the adaptable implementation package against usual practice on adherence to four different evidence-based ‘high impact’ indicators.*

11. Methods: The following points in the Methods differ from those described in the trial registry (ISRCTN registry ISRCTN91989345): In manuscript: “Audit and Feedback”, “Educational Outreach”, and “Prompts and Reminders” are described (Methods: Procedures: Lines 224-249). In the registered trial protocol, “GP Appraisal and Revalidation” and “Patient Mediated Intervention” are also described. Please explain, including in the manuscript.

We had originally set out to design and deliver a multifaceted intervention which included materials to help general practitioners when preparing for appraisal and revalidation, and materials to assist practices in their communication with patients concerning the targeted recommendations (patient mediated intervention). The feedback reports we delivered to practices did fulfil the former function of being used as supporting materials for appraisal and revalidation. As we report in a paper describing our delivered intervention (TIDIER checklist, Glidewell et al; reference 16), we had developed checklists to facilitate shared decision making with patients for managing blood pressure and diabetes but we could not make them available in a satisfactory format within the study timelines. We omitted this explanation from the original submitted manuscript to focus on the three main intervention components but have included it now in the revised manuscript.

12. Methods: The following points in the Methods differ from those described in the trial registry: There are a number of primary and secondary outcomes described in the manuscript (Methods: Outcomes: Lines 253-312). However, the registered protocol describes the primary outcome as: “Adherence; Timepoint(s): Adherence will be measures up to 12 mth post randomisation”, with no secondary outcomes described. Please clarify and explain the discrepancy. If the outcomes were not pre-specified in the protocol, please indicate that they were post hoc and explain why they were added. Post hoc comparisons should be presented as hypothesis generating rather than conclusive. Please explain, including in the manuscript.

Thank you for highlighting this further discrepancy. On the first point of the primary outcome, we did measure adherence to recommended practice for each of four targeted indicators, variously assessed through prescribing or attainment of recommended treatment goals. We did intend to assess these at 12 months after randomisation, originally scheduled for the end of March 2015, and over April 2015 to March 2016 to fit in with general practices’ data collection cycle for the Quality and Outcomes Framework. However, given circumstances beyond our control, we were unable to randomise until April 2015, and hence lost a month’s worth of post-randomisation data. We have now explained our original intention and the impact of the delayed randomisation on outcome assessment in the Methods and Results sections respectively. The secondary outcomes were not included when we first registered the trials but we did fully specify them in our published protocol (Willis et al., reference 19). All 11-month outcomes were therefore pre-specified and can be defined as conclusive rather than hypothesis-generating.

13. Methods: Line 328: Please provide a link to the pre-specified statistical analysis plan referred to here, and include as supplementary information.

A copy of the pre-specified statistical analysis is now included in the supplementary information as requested, and is referenced within the text.

14. Results: Line 347: Please provide statistical evidence (in the appropriate tables) for the statement that: “Baseline characteristics for general practices and patients were well balanced by trial and intervention (Tables 1 and 2).”

No statistical testing was used to assess for differences between arms for any of the baseline characteristics as recommended in the following publications:

1. Altman, D.G., Comparability of Randomised Groups. Journal of the Royal Statistical Society. Series D (The Statistician), 1985. 34(1): p. 125-136.

2. Senn, S., Testing for baseline balance in clinical trials. Stat Med, 1994. 13(17): p. 1715-26.

15. Results: Line 375: Please provide a reference for the statement: “The mean practice IMD was 30.22 (SD 13.86), within the top quarter of UK social deprivation.”

A reference for this statement has been added.

16. Results: Lines 429-441: For the following adjusted analyses (the main analyses describing effects of diabetes, risky prescribing, BP, and AF implementation package on outcomes) please also provide the unadjusted analyses.

The unadjusted analyses for the primary outcomes have been added to Table 3.

17. Results: Lines 445-447: For the following adjusted analyses (the analysis of the effect of risky prescribing implementation on adjusted prescribing levels) please also provide the unadjusted analyses.

The unadjusted analysis for the secondary outcome noted has been added to Supplementary Table 3.

18. Results: Lines 462-463: Please revise to: “Supplementary Figures 1-4 present cost-effectiveness planes and acceptability curves for the risky prescribing and blood pressure control implementation packages.” or similar.

This line has been amended as suggested.

19. Discussion: Line 541-543: Please provide an in-text reference to Supplementary Table 1 (S1 Table) for the statement that there are fewer eligible patients in the risky prescribing/anticoagulation AF arms.

This reference has been added.

20. Discussion: Please present and organize the Discussion as follows: a short, clear summary of the article's findings; what the study adds to existing research and where and why the results may differ from previous research; strengths and limitations of the study; implications and next steps for research, clinical practice, and/or public policy; one-paragraph conclusion.

Thank you for this advice. We have reviewed and revised our Discussion accordingly, taking reviewers’ comments into account (especially those of Reviewer 3).

21. Discussion: Thank you for including the link to the ASPIRE protocol, found at the end of the Discussion, after the Author Summary. It may be helpful to refer to any prospective study/analysis protocol in the Methods section of the manuscript. Also, the link does not seem to work. Please include the study protocol document and analysis plan, with any amendments, as Supporting Information to be published with the manuscript if accepted.

We apologise for the faulty link to the published protocol. We have now referred to and cited this in the Methods section of our manuscript. The pre-specified analysis plan has been included in the supplementary information and referred to within the Method section of our manuscript.

22. Figure 1: In the flow diagram, please indicate the number of individuals in each group analyzed in the ITT analysis. Please make it more clear in the diagram that the non-intervention control group (n=34) were not analyzed in the analyses being described here. This is written in the figure legend, but please include some visual indication in the diagram (text and/or shading) to make this point clear to the reader.

We agree that the flow diagram could be made clearer for readers. As suggested, the non-intervention control arm has been shaded, and the legend text revised, to clarify that the non-intervention control group are not analysed here.

We have also added the numbers of individuals in each group analysed in the ITT analysis, as requested. We had originally opted against including these as there are two values for each ‘Analysed’ box, corresponding to each of the trial arms. For example, in the diabetes control box we have total 1: the number of patients targeted by the diabetes control intervention, and total 2: the number of patients targeted by the risky prescribing intervention (since the diabetes control arm acted as the comparison for the risky prescribing arm). To aid readers, we have added explanation below the diagram.

We acknowledge that this adds complexity to the flow diagram and would welcome the views of the Reviewer and Editor as to whether these values should be included.

23. Figure 1: Please define the abbreviations: A&F (follow up).

Explanatory information has been added below the Figure.

24. Supplementary Figures 1, 2, 3, and 4 (S1, S2, S3, S4 Figures): Please define the following abbreviations: ICER, PSA, QALY, WTP.

We apologise for this oversight. The abbreviations have now been defined.

25. Table 3, and Supplementary Tables 3, 4, 5, and 6: Please provide the unadjusted comparisons as well as the adjusted comparisons.

The unadjusted analyses have been added to each of these tables as requested.

26. Table 3, and Supplementary Tables 3, 4, 5, and 6: Please specify the variables controlled for in the adjusted analyses in the Table legend.

The variables controlled for in the adjusted analyses are listed beneath each of the tables as requested.

27. Table 3: Please define the following abbreviations: CI, ICC.

This detail has been added

28. Supplementary Table 1: Please clarify sources of data in support of assumptions. Please explain the “mean achievement” measure in the table legend.

The data source for the sample size calculation and an explanation of the term “mean achievement” have been added beneath the table.

29. Supplementary Table 3 :Please define the following abbreviations: CI, BP, HbA1c, ACR, PCR, eGFR, BMI, NSAID, ACE-I, ARB, CKD.

This detail has been added.

30. Supplementary Table 4: Please define the following abbreviations: CI, QOF, IFCC-HbA1c, RCP, CHD, PAD, TIA, COPD.

This detail has been added.

31. Supplementary Table 5: Please define the following abbreviations: CI, BP, HTN, CKD, CHD, PAD, TIA, CVD, AF, CHA(2)DS(2)-VASc.

This detail has been added.

32. Supplementary Table 6: Please define the following abbreviations: CI, QOF, CHA(2)DS(2)-VASc, RCP.

This detail has been added.

33. Checklist: Please complete the CONSORT checklist and ensure that all components of CONSORT are present in the manuscript. Please include the completed checklist as Supporting Information (e.g. S1 Checklist). When completing the checklist, please use section and paragraph numbers, rather than page numbers.

The CONSORT checklist has now been revised, with section and paragraph numbers.

**Reviewer #1: Alex McConnachie**

Willis et al report on two cluster randomised trials of implementation packages delivered to general practices to improve diabetes control, risky prescribing, blood pressure control, and anticoagulation in AF. This review looks at the use of statistics in the paper.

Overall, the trial design, statistical methods, and presentation of results are very good. My one minor point is that it appears that the primary results were judged at a significance level of 2.5%; this is not explained in the statistical methods, but is mentioned in the results. I presume the reason for this is that each trial involved the assessment of two interventions, so a 2.5% significance level was use to preserve an overall 5% Type I error rate. This should be explained in more detail in the methods section.

However, it could also be argued that the significance level should be set at 1.25%, since the trial as a whole involved testing the effect of four interventions. On that basis, the effect of the risky prescribing intervention would not be considered statistically significant. Either way, with multiple primary analyses, a clearer description of how the authors controlled the false discovery rate should be given.

We used randomisation as a ‘fair’ way to assign general practices to one of two trials and promote balanced characteristics across the two trials. Once this assignment had occurred, both trials were independent of one another with no practices in Trial 1 contributing data to Trial 2 and vice versa. A lower significance level would have only been necessary had we used a 4-arm rather than a 2-arm design (which would have been unfeasible with the balanced incomplete block designs) testing a different hypothesis. We have added further clarification to the Methods section.

Was there a statistical analysis plan? Has this been published, or should it be provided as a supplement?

All analyses reported in the manuscript were carried out in accordance with a pre-specified and approved statistical analysis plan. The analysis plan has not been published. However, a copy is now included in the supplementary information as requested.

**Reviewer #2:**

This paper presents a trial that evaluates implementation science interventions for multiple evidence based indicators. I agree with the authors that this approach could be useful in the context of multiple morbidity.

However, the paper includes a number of flaws. The main ones are: the aim or hypothesis is not stated; the nature of the 'adaptation' included in the intervention is not sufficiently specified; the statistical results are not correctly interpreted; consequently, the conclusions may not be justified.

We respond to these points separately below.

Abstract: This could be clearer. In particular the sentence on randomisation would naturally follow-on from the sentence on recruitment

Thank you for picking this up. We have re-ordered the abstract to reflect the sequencing of methods in the study.

Introduction: This is clear and succinct. More could have been made of multiple morbidity. The study appears to lack a clear aim or hypothesis. The final paragraph here, explains what was done but not the purpose.

We aimed to keep the Introduction fairly concise. We could have made more about multiple morbidity but the main driver behind the study is that practices need to respond to multiple guidelines. We have specified the study objective, including comparison, as mentioned earlier.

Methods: The study design could be explained more clearly. Initially, it says that this is 'two parallel cluster randomised trials using balanced incomplete block designs'. This should be explained more completely: what are the intervention and comparator trial arms in each trial. Why does this represent a balanced incomplete block design?

We agree that we could have explained and illustrated this study strength more clearly. We have therefore included the rationale for the balanced incomplete block design in the methods and provided an example of how the comparison worked.

Later it refers to 'two stage randomisation' which suggests a single trial, the written description is not clear though Figure 1 is. Looking at Figure 1, the design is clear but the research question is not.

We have clarified both the randomisation process and the research question as recommended above.

It appears that the no intervention control trial arm was not included in the protocol but was only added because recruitment exceeded expectations. The paper then says that the control group analysis will not be reported in this study. It may be best to focus this paper on what was in the protocol only.

We agree that describing the no-intervention arm does increase the complexity of the paper. However, we are obliged to mention it in order to explain the flow of practices in the CONSORT diagram and because it was pre-specified in the protocol. We have clarified that analysis of this additional group is not the main focus of the paper.

The software for the minimisation could be mentioned. Also, how allocation was concealed as distinct from blinding.

The software used for the minimisation has been added in the ‘randomisation and masking’ section of the manuscript. Practice and trial personnel were not blind to allocation; this has been clarified in the abstract and in the methods.

The description of the intervention is generic. It is not made clear how the intervention package differed for each of the four trial arms. What are the comparisons being made? The title refers to an 'adaptable' intervention though the basis of adaptation is not mentioned in the methods.

We agree that the nature of the adaptation should have been made clearer. We have made this more explicit in the Methods:

*Implementation package content, specifically the clinical content and embedded behaviour change techniques, was adapted for each of the four targeted indicators…*

Under outcomes it refers to 'primary outcomes for each intervention arm'. Normally there is one primary outcome for a trial. Here there are four intervention arms, but the study comprises two trials. Multiple comparisons require additional discussion.

This paper describes the methods and results of two cluster randomised trials. As the trials utilised a balanced incomplete block design and evaluated two interventions per trial, we inherently have two primary outcomes per trial. Within each trial, we adjusted for multiple comparisons as explained under Sample Size. Further clarification has been added to Statistical Analysis.

Missing data: initially it says that missing data could not be assessed, then it says that data were assumed missing at random. Does it mean that not recorded values were assumed to be not performed for process items? Was imputation performed for values like blood pressure? or were complete case analyses performed it is not clear.

The statement that missing data could not be assessed refers to the fact that the clinical indicator outcomes are based on combinations of values and diagnosis codes present in the patient electronic health record. There is no method to identify if a particular code is not recorded where it should be as we have received the routine data only. We are assuming that any missing diagnosis codes are missing at random within the electronic health record data. The text in the statistical analysis section has been updated to provide more detail.

Imputation was not performed for the continuous clinical outcomes. It has now been explicitly stated that a completed case analysis was undertaken in these cases. We specifically mention continuous outcomes here as we were able to identify patients with missing values (of those who were eligible for the primary outcomes). In contrast, for the binary indicator outcomes which were extracted from SystmOne, all of the outcomes were either yes/no but never missing. There may have been missing data on SystmOne (missing read codes) that led to incorrect classification as Yes/No, but we had no way of assessing whether that was the case.

Results: Page 24 lines 429 and 436 and 439, where it says 'no effect', 'absence of evidence is not evidence of absence' as Doug Altman pointed out. <https://www.bmj.com/content/311/7003/485>

We agree with this nuanced point and have amended the wording to ‘no observed effect’.

line 434, where it says 'statistically significant improvement. The paper should interpret P values following the ASA recommendations. Cut points such as P<0.05 or P<0.025 should not be used. Reporting should be based on ASA guidelines with not 'bright lines' for interpretation such as P<0.025. <https://amstat.tandfonline.com/doi/full/10.1080/00031305.2016.1154108#.XVpvSuhKiUk>

The ASA recommendations oppose the sole use of a p-value as strict evidence against a proposed hypothesis. We agree with this and understand the need for more cautious language around the statistical significance of our primary and secondary outcomes. The use of a p<0.025 cut-off for the primary outcomes is in accordance with our pre-specified statistical analysis plan, which details a sample size calculation based on a 2.5% significance level (adjusting for two outcome comparisons per trial). However, the wording of our conclusions has been amended in respect of the fact that the p<0.025 is not truly a definitive measure of a successful intervention.

Similarly line 442 where it says 'no statistically significant effect'

The text has been updated in accordance with the response above.

Line 443 where it says 'significantly improved one of the indicators', was adjustment made for multiple comparisons?

We did not adjust for multiple comparisons on the secondary outcomes and this has been clarified in the Methods. We acknowledge that this increases the chances of reporting a spurious finding. There is accordingly less emphasis on the secondary outcomes in the results section of the paper. For the indicator noted as ‘significant’, we have amended the wording to a more cautious interpretation and refer to this in the Discussion.

In general insufficient details are provided for the cost-effectiveness analysis.

Line 458 'the deterministic ICER for the BP packages indicates cost-effectiveness'. How can this be so when the intervention had 'no effect' (line 436).

The health economic component is substantive, not least because it includes three evaluations and four decision models. Given this, we would not be able to include anything approaching the full details here. We hope to do this in a separate publication.

Re: line 458. We have amended the earlier line (previously 436) to indicate that there was no observed effect of the intervention on blood pressure control. Despite this, given that the results were in the positive direction the economic evaluation indicated cost-effectiveness (although, of course, this was highly uncertain).

Discussion

Again, the paper concludes that the intervention package had no effect for three out of four indicators, but this could be an 'absence of evidence is not evidence of absence error'. Consequently, the conclusion may be too strong. Perhaps more evidence will show that an adaptive approach may be effective.

We have now stated that there was ‘no observed effect.’

The paper arrives at the conclusion that an adaptable intervention 'does not work' but it is not made clear from the analysis plan how the various comparisons that were made could lead to such a conclusion.

We have amended the wording in the Author Summary as described above. We agree that it would be erroneous and premature to conclude with confidence that the adaptable implementation package did not work on the basis of only four comparisons. Indeed, we have never been quite convinced by the often used cliché in implementation research that ‘one size does not fit all’ – which underpinned some of the motivation behind our programme of work. Therefore, we have removed any wording which implies this and focused instead on offering explanations for the differences we observed in the four primary outcomes.

**Reviewer #3:**

This manuscript describes the findings from a large implementation trial intended to improve quality in primary care for 4 common conditions. The authors went through a careful process of selecting performance measures that matter and designing an implementation package based on the best available evidence at the time. They then implemented this in a large number of GP practices, and assessed the fidelity of the intervention. No differences were seen in their primary outcomes except a decrease in what they term "risky prescribing", meaning a patient safety issue, from 6.0% to 4.9%. A cost effectiveness analysis showed that for this one target, their intervention was cost-effective. The authors conclude that "a one size-fits-all implementation package only worked for 1 in 4 indicators".

The topic area of this manuscript - improving quality and safety in primary care - is high on the agenda for all health care systems. As someone who regularly reviews quality improvement publications as part of their scholarly activities, I can say with authority that this manuscript is a very high outlier in terms of the preparation and planning that went into the intervention and the way it is reported.

Note I am not a cost-effectiveness analyst and so can make no informed critique of their methods or results in this regard.

The results of the QI process must have come as a big disappointment to the investigators, and will be disappointing to readers as well, but these are results that have to be widely disseminated in order for ongoing and planned implementations to be changed now, to avoid continuing ineffective QI initiatives.

Thank you for this feedback. Whilst we may also have been a little disappointed by the results, we are satisfied that we completed a pair of trials which maximised both rigour and pragmatism.

I have only a few comments for the authors to consider, all in the discussion sections about "Why?" The first and most obvious is already touched on briefly by the authors, namely that of their primary outcomes, the risky prescribing measure is the easiest for a GP to do. For two other conditions the performance measure is an intermediate outcome (or even a composite of several intermediate outcomes), and therefore much harder for the GP to influence. The AF indicator is a process measure but one that requires 1) that the GP calculate the CHADS-VASC score (maybe the EHR already does this automatically - if so then the manuscript should make this point) AND 2) prescribe a medication known to have a certain risk to it, plus be a pain-in-the-rear-end for patients and practices to monitor: they are signing themselves up to regular monitoring of INR, unless they are going with a new oral agent, which i suspect at the time this trial was going would have been rare in UK GP practices. As opposed to the "risky prescribing" measure, which only required someone to choose paracetamol instead of an NSAID or add omeprazole if they go with the NSAID. So, I wonder if the authors want to discuss a little more the 'easiness' of meeting this measure as opposed to the others. I understand that the process measures for diabetes - also easy things to do - didn't have significant improvements in their intervention group (although they all were a little better in the intervention vs the control), but most of them are topped out, anyway, with not a lot of room for improvement. So I think there is more-than-a-hint that these data are compatible with the conclusion that influencing a process measure is easier than influencing an outcome measure. Perhaps the authors want to expand a little on this text in the discussion, right now it is two sentences and, to me, focuses on the wrong thing- namely that BP control requires 2 visits - instead of the much-more-difficult problem of finding a pharmaceutical that is both effective and acceptable to the patient. The current text also does not deal with the AF performance measure, which like the risky prescribing measure only requires the GP to add a medication. Why isn't adding warfarin as easy as adding omeprazole? Understanding why is key to understanding why their intervention had an effect for one and did not for the other.

Ironically, when we went through an earlier process of screening a total of 2365 clinical practice recommendations and selecting a small number for development as ‘high impact’ indicators, our selection criteria included ‘the extent to which following a recommendation is directly within the control of individual practice teams or professionals.’ We now welcome the opportunity to share our experience and caution others with hopeful expectations.

We have followed the Reviewer’s advice and elaborated more on why we observed an improvement in risky prescribing but not in other indicators.

Secondly, I wonder whether the "patient safety" angle of the "risky prescribing" measure played a role. The other measures are all clinical quality, whereas the risky prescribing measure is a patient safety issue, making it perhaps more important in the GP's eyes.

We agree with this suggestion and have added a comment to the Discussion.

Thirdly, I think a little more information about the EHR prompt is warranted. Have I concluded correctly that this was the only measure that had an EHR prompt included? And was the prompt a hard stop, requiring an over-ride, or a soft-stop, something easily blown through by the GP? Judging by the size of the effect, I am assuming a soft stop. These data are also consistent with a conclusion that EHR prompts work for simple actions, which the authors note, but some more information about the prompt, and why there wasn't a prompt for the warfarin measure, would help readers understand when a prompt might work (I am presuming this is because the NSAID prompt was triggered by the e-prescribing of an NSAID, which the EHR can then assess against age, concomitant cytoprotection, GFR, and concomitant warfarin, whereas the AF measures would require an alert to be triggered by something else much more complex. The authors cite another trial in this regard, but rather than make readers go look up that trial they would benefit from a sentence or two about what makes one easy and the other complex).

‘Risky prescribing’ may not always be inappropriate and we therefore decided against enforcing a ‘hard stop’ in the electronic health record to allow scope for individual clinical judgement. However, the prompt still triggered a one-step justification to continue prescribing. We have mentioned this in the Discussion.

Lastly, there is the question of additional incentives. When practices are already being paid via QOF for doing a host of things, how much mental energy is left over to participate in something where they aren't getting more payments for doing the "thing"?

We also agree that this issue merits further consideration and have commented on it further.

So...bottom line, an important study with important (but disappointing) findings, that in my view would benefit from a couple hundred extra words from the authors speculating about why they think some aspects did and did not work as expected

Thank you. We are currently writing up a process evaluation to shed further light on the findings from the trials.
