# Supplementary material for: An adaptable implementation package targeting evidence-based indicators in primary care: A pragmatic cluster-randomised evaluation
Source: PLoS Med. 2020 Feb 28;17(2):e1003045. doi: 10.1371/journal.pmed.1003045 (PMC7048270; doi:10.1371/journal.pmed.1003045)
Supplement: Attachment — Submitted filename: PMEDICINE-D-19-02682R1 response to reviewers 200120.docx [file pmed.1003045.s015.docx]

Response to reviews

**An adaptable implementation package targeting evidence-based indicators in primary care: a pragmatic cluster-randomised evaluation**

**PMEDICINE-D-19-02682R1**

We thank the editors and reviewers for helpful feedback on this manuscript. We believe that we have now been able to improve the manuscript in light of this feedback.

**Requests from Editors**

1. Competing Interest Statement: Thank you for clarifying the roles of the authors in the updated competing interest section in your manuscript. Please update the competing interest statement on the manuscript submission form.

Apologies for failing to update this section on the manuscript form. The statement has now been updated.

2. Data Availability Statement: Thank you for providing the contact information for access to your study data. We suggest you shorten the Data Availability Statement to: “Data cannot be shared publicly owing to a need to main patient confidentiality. Interested researchers may contact [ctru-dataaccess@leeds.ac.uk](mailto:ctru-dataaccess@leeds.ac.uk) to request and obtain relevant data.” Please update this in the manuscript submission form with your revised submission.

Thank you for this suggestion. The statement has been revised, in both the manuscript and on the submission form.

3. Throughout: Abstract Lines 104 and 112, Introduction Line 198, and Methods Line 239: Please replace the term “usual practice” with “implementation control”, as your control comparison condition is not accurately described as usual practice.

The change has been made throughout.

4. Throughout:Trademark (™) symbol: Please remove the trademark symbol throughout the manuscript (Lines 218, 309, 363, 367, 396).

This has been removed throughout.

5. Abstract: Line 104-105: In the final sentence, please clarify the nature of the indicators (indicators of what?)

The sentence now reads: “…evidence-based quality indicators”.

6. Abstract: Line 108-109: Please revise this sentence to: “We used ‘opt-out’ recruitment, and practices that did not opt out were randomly assigned to an implementation package…”

This sentence has been revised.

7. Abstract: Line 123-124: Please describe the confounds for which you adjusted.

We have added additional detail about the confounders we adjusted for. This had the consequence of taking the Abstract over the 500 word limit and so we have made some further edits to bring the total back below the limit.

8. Abstract: Line 127: Rather than saying that the implementation package “had no effect on other primary endpoints”, please provide the adjusted ORs ( with CIs and p values) for all four primary comparisons from Table 3.

This detail has now been added for all four primary comparisons.

9. Abstract: Conclusions: Your conclusion only touches on the cost-effectiveness of the intervention. Can you please include your main conclusions regarding the primary endpoint (i.e. adherence is your registered primary endpoint, and the description above in the abstract describes specific patient-level primary endpoints that could be discussed). At Line 135, please avoid using the term “relatively cost-effective”; instead, please use quantitative terms to describe cost-effectiveness.

Thank you for this comment. We would welcome further advice from the Editorial team on this point. Both the primary outcome finding and the cost-effectiveness result are reported in the Results section of the Abstract and we had attempted to interpret and summarise these in an efficient manner in the Conclusion. It is our view that it would be unnecessary to repeat both here.

We have amended the sentence to demonstrate that the intervention was both clinically and cost-effective; we have deleted “relatively” from the description of cost-effectiveness.

10. Author Summary: “What did the researchers do and find?”: Please remove the word “highly” from the first bullet point. Also, please clarify if you are reporting results of one trial or two trials, and mention the outcomes being assessed.

The word “highly” has been removed, as requested. We have also clarified that the paper reports on two trials, and mentioned the primary endpoints.

11. Author Summary: “What do these findings mean?”: In the first point, we suggest you also mention your conclusion regarding the primary outcome of the study, in addition to mentioning the cost-effectiveness outcome.

Please see our response to point 9, above.

12. Methods: Please add the following statement to the Methods section: “This study is reported as per the Consolidated Standards of Reporting Trials (CONSORT) guideline for cluster randomized trials (S1 Checklist).”

This sentence has been added to the Methods section, at the end of ‘Study design and participants’.

13. Discussion: Lines 532-533: Please clarify the second part of this sentence, we suggest: “...which could ultimately be associated with changes in mortality, morbidity and health service use, depending on generalizability in the general population.” or similar.

The sentence has been amended, as suggested.

14. Discussion: Lines 550-551: Please clarify this sentence: “The risky prescribing package cost of £0.28 per patient is inexpensive.” Inexpensive relative to what?

This sentence has been revised as follows:

*“It is noteworthy that the cost of the risky prescribing package was only £0.28 per patient.”*

15. Discussion: Line 558: Please remove the word “highly” from the sentence.

The word has been removed.

16. References: Please double check that all references are formatted with the "Vancouver" style for reference formatting, and see our website for other reference guidelines <https://journals.plos.org/plosmedicine/s/submission-guidelines#loc-references>

For example, reference 7 should be “PLoS ONE”, ref 18, 28, 32, are not complete/have extraneous text; ref 21, 27, should be N Engl J Med.

Thank you for providing the link to the guidance on reference formatting, we apologise for the errors in the previous manuscript. We believe that all References are now formatted correctly.

17. Link to Trial Protocol: Line 649-650: Thank you for including the trial protocol with your supporting information. The Supporting Information will be available online with the manuscript. As you have included the protocol document, you can remove the extra link here.

The link has been removed.

18. Table 2: Please check the “a” “b” “c” superscripts in the table footnote, there appears to be some discrepancy.

Thank you for identifying this error. The superscripts have now been allocated correctly.

19. Supplementary Figure 2 and 4: Please integrate the “thousands” label for the X axis units with the X axis legend.

The label has been amended as requested.

20. Checklist: Thank you for including your CONSORT checklist. For Item 1a, please change the location to “Title” and for item 1b, please change the location to “Abstract”. For item 6b, you could direct the reader to where you discuss changing your primary outcome assessment timepoint to 11 months rather than 12 months. For item 24, please reference your trial protocol included in the supporting information (e.g. S1 text). For item 25, please reference “Funding” as the section for this information.

The CONSORT checklist has been updated to incorporate all of these points.

**Additional amendment**

We wish to make a further, minor change to the Discussion that is not in response to any of the editorial/reviewer comments. While proofreading the manuscript, we felt that line 626/627 (marked version) was potentially confusing and did not accurately reflect the analysis conducted. We have edited it to read as follows:

“The observed significant difference in achievement of the associated secondary outcome on gastro-protection provides evidence to support such a mechanism (although we would still advise cautious interpretation given that these analyses were not formally adjusted as we tested across multiple secondary endpoints).”

**Comments from Reviewers**

**Reviewer #1**

Having reread the paper, and the authors' responses to my and the other reviewers' comments, I am happy with the changes that have been made. I have no further comments to make.

We again thank the Reviewer for their time and contribution to our paper.

**Reviewer #2:**

The revision has generally responded to the review comments.

In the Abstract where it reads: 'The implementation package reduced risky prescribing (odds ratio 0.82; 97.5% confidence interval (CI) 0.67 to 126 0.99, p = 0.017) with an incremental cost effectiveness ratio of £1,359 per quality-adjusted life year but had no effect on other primary endpoints. No statistically significant effects were observed in any secondary outcome except for reduced co-prescription of aspirin and clopidogrel without gastro-protection in patient aged 65 and over (adjusted OR 0.62; 97.5% 130 CI 0.39 to 0.99; p = 0.021). '

It appears that the abstract is reporting only those results that are associated with P values less than 0.025, whereas the other outcomes are referred to as having 'no significant effects'. It would be preferable to report each of the primary outcomes in the Abstract.

The ASA guidelines on P values make the recommendation that 'Scientific conclusions and business or policy decisions should not be based only on whether a p-value passes a specific threshold.' The authors justify their approach with respect to the analysis plan, but it would be preferable to follow the ASA recommendation. Rather than saying there was 'no effect', there was 'insufficient evidence of effect', I expect the authors do not want to add to the catalogue of 'absence of evidence is not evidence of absence' errors.

Thank you for these further comments. The full primary outcome results are now presented in the Abstract (as was also requested by the Editors). We have also rephrased the lines in the paper that referred to there being ‘no effect’ of the intervention upon three outcomes.

**Reviewer #3:**

I don't have any remaining issues with this manuscript and would proceed with accepting it.

We again wish to think the Reviewer for their time and contribution to our paper.
